# Global, regional, and national burden of bone and joint infections, 1990–2021: a comprehensive analysis of trends, pathogens, and antimicrobial resistance

**DOI:** 10.3389/fcimb.2026.1858745

**Published:** 2026-06-02

**Authors:** Ke Xu, Xia Zhao, Shuangqing Zhang

**Affiliations:** 1 Department of Operating Room, The First Affiliated Hospital of Chengdu Medical College, Chengdu, Sichuan, China; 2 Department of Orthopedics, The First Affiliated Hospital of Chengdu Medical College, Chengdu, Sichuan, China

**Keywords:** antimicrobial resistance, bone and joint infections, burden, DALYs (disability adjusted life years), *Staphylococcus aureus*

## Abstract

**Background:**

Bone and joint infections (BJIs) are disabling conditions whose global burden is evolving. This study aimed to systematically analyze the disease burden, temporal trends, pathogen spectrum, and resistance profiles of BJIs at the global, regional, and national levels from 1990 to 2021.

**Methods:**

Based on the Global Research on Antimicrobial Resistance Project, this study extracted data on deaths and disability-adjusted life years (DALYs) attributable to BJIs from 1990 to 2021, stratified by sex, age, region, pathogen, and antimicrobial resistance. Trends and inequalities were analyzed using estimated annual percentage change, the Bayesian age-period-cohort model and health inequality analysis.

**Results:**

Between 1990 and 2021, the global number of deaths due to BJIs increased by 228% (from 7,475 to 24,505), and DALYs increased by 291% (from 1,549,957 to 6,054,611), with age-standardized rates showing a consistent upward trend. The disease burden exhibited significant geographical inequality, with East Asia bearing the heaviest and fastest-growing burden. *Staphylococcus aureus* was the leading pathogen responsible for both mortality and DALYs. Regarding antimicrobial resistance-associated burden, the death toll attributable to antimicrobial resistance increased markedly, with *Staphylococcus aureus*, *Escherichia coli*, *Pseudomonas aeruginosa*, and *Klebsiella pneumoniae* being the most prominent contributors.

**Conclusions:**

The global burden of BJIs continues to increase, exhibits significant regional inequalities, and antimicrobial resistance serves as a critical driver of health loss. These findings necessitate strengthened infection prevention and control, prudent antimicrobial use, and the development of novel therapeutic strategies.

## 1 Introduction

Bone and joint infections (BJIs) refer to a group of infectious diseases caused by pathogens invading the bones, joints, and surrounding tissues, manifesting as local pain, functional impairment, and bone destruction, often progressing to a chronic state. In severe cases, they may develop into systemic infections, sepsis, and even lead to disability. These infections not only have a relatively high incidence but also impose a significant risk of disability and a substantial medical burden on both patients and society (Malizos and Kirketerp-Møller, 2016; Liu et al., 2025). The causative pathogens are diverse, including bacteria, fungi, and mycobacteria, with bacterial infections being the most common. In recent years, the emergence and spread of antimicrobial resistance have further complicated the diagnosis and treatment of BJIs (Salarvand et al., 2023; Useinova et al., 2023). Moreover, armed conflicts and wars constitute an important yet frequently overlooked driver of BJIs and antimicrobial resistance, as military injuries, blast-related open fractures, and contaminated wound environments create conditions highly conducive to BJIs, while the disruption of healthcare infrastructure in conflict zones severely undermines infection prevention, antimicrobial stewardship, and access to definitive surgical care, thereby facilitating the emergence and spread of resistant organisms (Pallett et al., 2023; Pitiot et al., 2025).

Globally, factors such as an aging population, the rising prevalence of chronic diseases, increased volume of orthopedic surgeries, higher rates of trauma, and inappropriate use of antimicrobial agents are expected to exacerbate the disease burden of BJIs, posing serious challenges to public health systems (Zhang et al., 2024). Multiple studies have shown that BJIs often occur as complications of orthopedic surgeries or result from hematogenous spread, representing a major source of infection among hospitalized patients and a key risk factor for long-term disability and mortality (Sara et al; Naruse, 2018). Although existing international cohort studies provide valuable references, their data are largely derived from high-income countries or cross-sectional designs, lacking systematic longitudinal data that span different geographical regions, economic levels, and time dimensions (Subedi et al., 2025). This knowledge gap is particularly critical: despite continuous advances in surgical techniques, antimicrobial therapy, and infection control measures, the disability and mortality rates associated with BJIs have not been fundamentally improved, which suggests that regional variations in pathogen profiles, area-specific resistance patterns, and differences in diagnostic and therapeutic strategies across healthcare settings may be important factors hindering the improvement of prevention and treatment outcomes.

Currently, global epidemiological data on BJIs remain insufficient, especially regarding the dynamic evolution of pathogen composition and antimicrobial resistance characteristics, for which long-term and systematic assessments are still lacking. Therefore, to address this knowledge gap, this study aims to systematically analyze the disease burden, temporal trends, and pathogen profiles along with resistance characteristics of BJIs at the global, regional, and national levels from 1990 to 2021, with the goal of providing a scientific basis for improving related public health strategies and achieving targeted prevention and control.

## 2 Methods

### 2.1 Data sources

This study is based on the Global Research on Antimicrobial Resistance (GRAM) Project, a collaboration between the University of Oxford and the Institute for Health Metrics and Evaluation at the University of Washington (GBD 2021 Antimicrobial Resistance Collaborators, 2024) Building upon the Global Burden of Disease Study analytical framework, the GRAM Project integrates multiple data sources, including vital registration systems, mortality surveillance records, hospital discharge datasets, outpatient and emergency department insurance claims, microbiological laboratory data, and systematic literature reviews, estimating the mortality burden for 12 infectious syndromes, 64 pathogens (encompassing 22 antimicrobial-resistant bacteria), and 84 pathogen-drug combinations (GBD 2021 Antimicrobial Resistance Collaborators, 2024; Kariuki, 2024).

The GRAM Project’s estimation framework follows a multi-step analytical pipeline (GBD 2021 Antimicrobial Resistance Collaborators, 2024). First, the fraction of sepsis-related deaths for each underlying GBD cause was estimated using mixed-effects binomial logistic regression, with the Healthcare Access and Quality Index and sex included as covariates and a nested random effect on underlying cause of death. Second, sepsis-related deaths were apportioned to multiple infectious syndromes using an informative ranking hierarchy based on ICD-coded diagnoses, ensuring the most pathogen-informative syndrome was prioritized when multiple syndromes co-occurred. Third, pathogen distributions within each syndrome were estimated using spatiotemporal Gaussian process regression (ST-GPR) models incorporating multiple data sources. Fourth, the prevalence of antimicrobial resistance for each pathogen-drug combination was modeled using standardized resistance breakpoints with ST-GPR, accounting for covariates including antibiotic consumption. Fifth, the relative risk of death associated with resistant versus susceptible infections was estimated from matched cohort data. Finally, a counterfactual framework was employed to compute both associated (all deaths involving resistant infections) and attributable (excess deaths due to resistance) antimicrobial resistance burden.

In the GRAM framework, BJIs are classified under the infectious syndrome “Infections of bones and joints “ which is one of 12 infectious syndromes contributing to the estimated antimicrobial resistance burden (Antimicrobial Resistance Collaborators, 2022; GBD 2021 Antimicrobial Resistance Collaborators, 2024). This syndrome encompasses acute osteomyelitis and infectious (septic) arthritis affecting the bones, joints, and adjacent musculoskeletal structures, while chronic osteomyelitis and Lyme arthritis were excluded from the etiological data inputs. BJIs were identified through a multi-step mapping process: International Classification of Diseases (ICD) codes recorded in multiple causes of death data, hospital discharge records, and linked mortality datasets were mapped to the GBD cause hierarchy, and deaths involving infection were subsequently classified into infectious syndromes using an informative ranking algorithm that prioritizes the syndrome with the most distinctive pathogen distribution (Antimicrobial Resistance Collaborators, 2022). The syndrome-specific burden was estimated using mixed-effects binomial logistic regression models, with the Healthcare Access and Quality Index and sex as covariates, stratified by 13 age groups (Antimicrobial Resistance Collaborators, 2022). Notably, BJIs are operationalized as an infectious syndrome rather than an independent GBD cause of death, and the GRAM framework does not provide further clinical subcategorization (e.g., prosthetic joint infections, spondylodiscitis, or periprosthetic infections), which constitutes a recognized limitation of the administrative data-based approach.

All estimates are derived from 500 iterations, with final results presented as the mean and its 95% uncertainty interval (UI), where the 95% UI is defined by the 2.5th and 97.5th percentiles of the 500 iterations. Acknowledging that the GRAM Project’s statistical models synthesize data subject to inherent limitations—such as variations in geographical and temporal data coverage, diagnostic practices, and reporting completeness—the study employs standardized methods, bias correction, and covariate-based extrapolation for adjustment. The methodological framework is detailed in previous publications (GBD 2019 Antimicrobial Resistance Collaborators, 2022; GBD 2021 Antimicrobial Resistance Collaborators, 2024).

In this study, we extracted data on BJIs from 1990 to 2021, including death and disability-adjusted life year (DALY) estimates, stratified by sex, age, and region, focusing on the burden attributable to different pathogen profiles and the burden due to antimicrobial resistance.

The data used in this study are publicly accessible, and therefore, the Ethics Committee of The First Affiliated Hospital of Chengdu Medical College determined that ethical approval was not required. The study adheres to the guidelines for cross-sectional studies outlined in the Guidelines for Accurate and Transparent Health Estimates Reporting (Stevens et al., 2016).

### 2.2 Model validation of GRAM-derived estimates

The GRAM Project has employed multiple validation approaches to assess model performance. For the infectious syndrome classification models, out-of-sample 5-fold cross-validation was performed, yielding high accuracy (range: 0.87–1.00) and AUC scores (range: 0.50–0.98) across syndrome-age group combinations, with notably strong performance for bloodstream infections (AUC: 0.87–0.98) and lower respiratory infections (AUC: 0.73–0.94). The pathogen distribution models were validated using root mean square error and mean absolute error metrics against held-out data. The prevalence of resistance models were evaluated using AUC analyses comparing predicted versus observed resistance profiles (GBD 2021 Antimicrobial Resistance Collaborators, 2024).

### 2.3 Statistical analysis

In this study, both the age-standardized mortality rate (ASMR) and age-standardized disability-adjusted life year rate (ASDR) are expressed per 100,000 population. Additionally, we analyzed the correlation of the socio-demographic index (GBD 2019 Demographics Collaborators, 2020) with ASMR and ASDR. Advanced statistical methods, including estimated annual percentage change (EAPC) (Hankey et al., 2000), the slope index of inequality (Hosseinpoor et al., 2015), the concentration index (Ordunez et al., 2019), and the Bayesian age-period-cohort (BAPC) model (Fosse, 2020), were employed to systematically examine the distributional inequality, temporal trends, and driving factors of the disease burden. The BAPC model decomposes the log-transformed mortality rates into additive age, period, and cohort effects. A second-order random walk prior was imposed on each dimension, assuming that the second differences of adjacent parameters follow a normal distribution, thereby enforcing temporal smoothness while allowing for data-driven variation. The model employed an intrinsic Gaussian Markov random field specification under the integrated nested Laplace approximation framework for computationally efficient Bayesian inference. For the forecasting period, the period and cohort effects were extrapolated based on the trajectory estimated from historical data, with the caveat that the variance of projections increases with the length of the forecast horizon. All analyses and visualizations were performed using R software (v.4.3.1).

## 3 Results

### 3.1 Global trends

**Figure 1:**
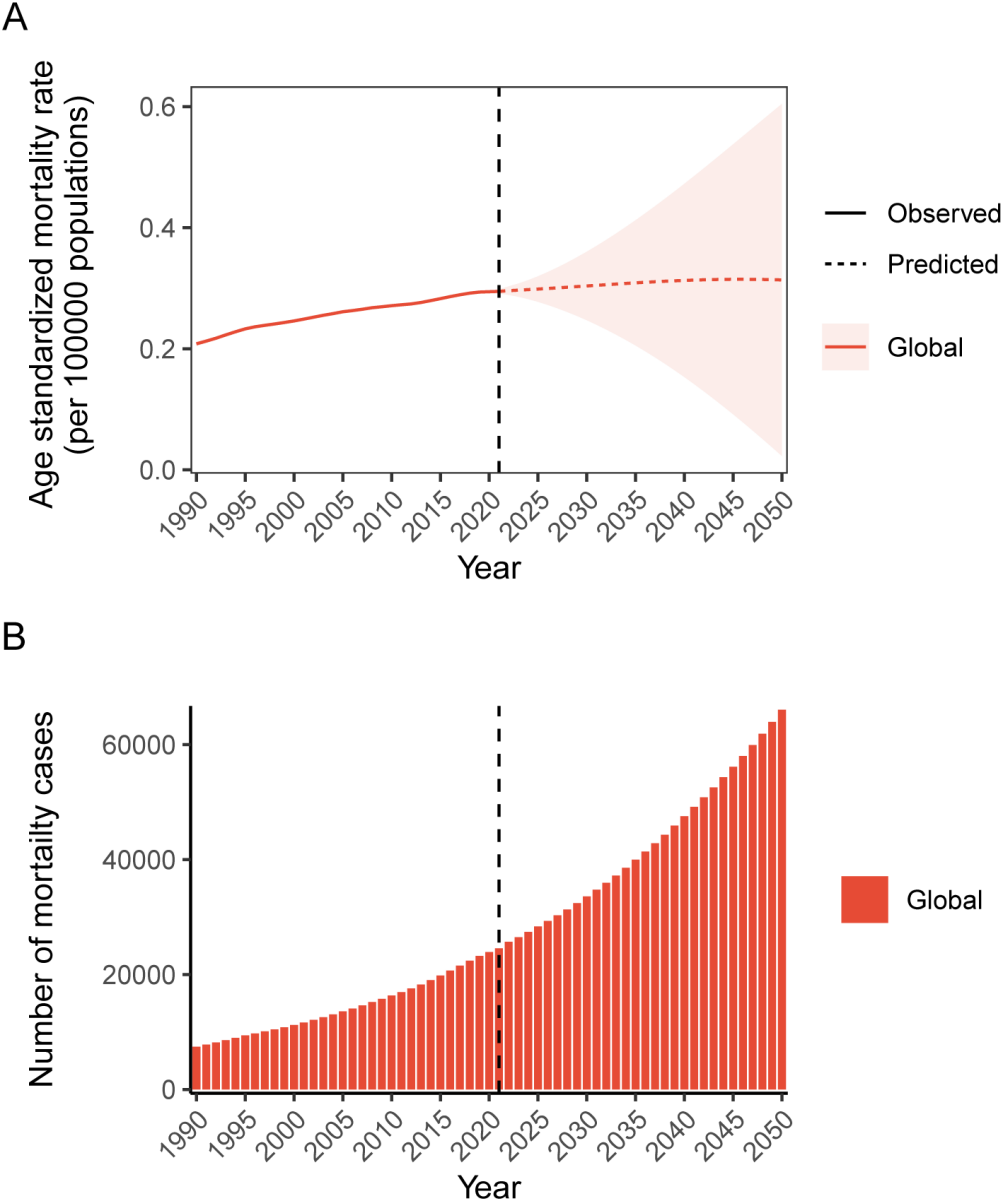
Future forecasts of age-standardized rates of mortality **(A)** and mortality number **(B)** in bone and joint infections from 2022 to 2050.

From 1990 to 2021, the global number of deaths due to BJIs increased from 7,475 to 24,505, representing a rise of 228%. The ASMR increased from 0.21 to 0.29 per 100,000 population. During the same period, DALYs attributable to BJIs increased from 1,549,957 to 6,054,611, an increase of 291%, while the ASDR rose from 38.84 to 71.53 per 100,000 population (**Supplementary Figure 1**; **Supplementary Table 1**). The EAPC in the global ASMR was 1.06%, and that for ASDR was 1.94% during this interval (**Supplementary Table 1**). Based on the BAPC model, it is predicted that by 2050, the global number of deaths due to BJIs will reach 66,080, with an ASMR of approximately 0.31 per 100,000 population (**Figure 1**).

### 3.2 Regional discrepancies

From 1990 to 2021, all regions experienced an increasing trend in the ASMR and ASDR for BJIs, with East Asia exhibiting the most pronounced increase. The EAPCs in this region were 3.74 for ASMR and 5.81 for ASDR (**Supplementary Figure 2**; **Supplementary Table 1**). In 2021, East Asia carried the highest burden of BJIs, with 5,289 deaths and 1,432,594 DALYs.

### 3.3 National variations

In 2021, there were significant disparities in the ASMR of BJIs across 204 countries and territories, ranging from 0.12 per 100,000 in Tajikistan to 1.13 per 100,000 in Bahrain. In terms of trends, Egypt experienced the fastest increase in ASMR (EAPC = 4.37), while the Democratic People’s Republic of Korea showed the steepest decline (EAPC=–1.34). Meanwhile, the ASDR varied from 13.90 per 100,000 in Tajikistan to 208.11 per 100,000 in Qatar. China recorded the most rapid rise in ASDR (EAPC = 6.28), whereas Burundi had the largest decrease (EAPC=–1.56) (**Supplementary Figure 3**; **Supplementary Table 1**).

### 3.4 Age trends and gender disparities

In 2021, the global number of deaths due to BJIs peaked in the 70–74 age group, with 3,373 cases, while the mortality rate increased steadily with age, reaching a peak of 11.07 per 100,000 in the ≥95 age group. The age distribution pattern of DALYs was generally consistent with that of mortality. Furthermore, the burden of BJIs was generally higher in males than in females (**Supplementary Figure 1**; **Supplementary Table 2**).

### 3.5 Correlation with the sociodemographic index

**Figure 2:**
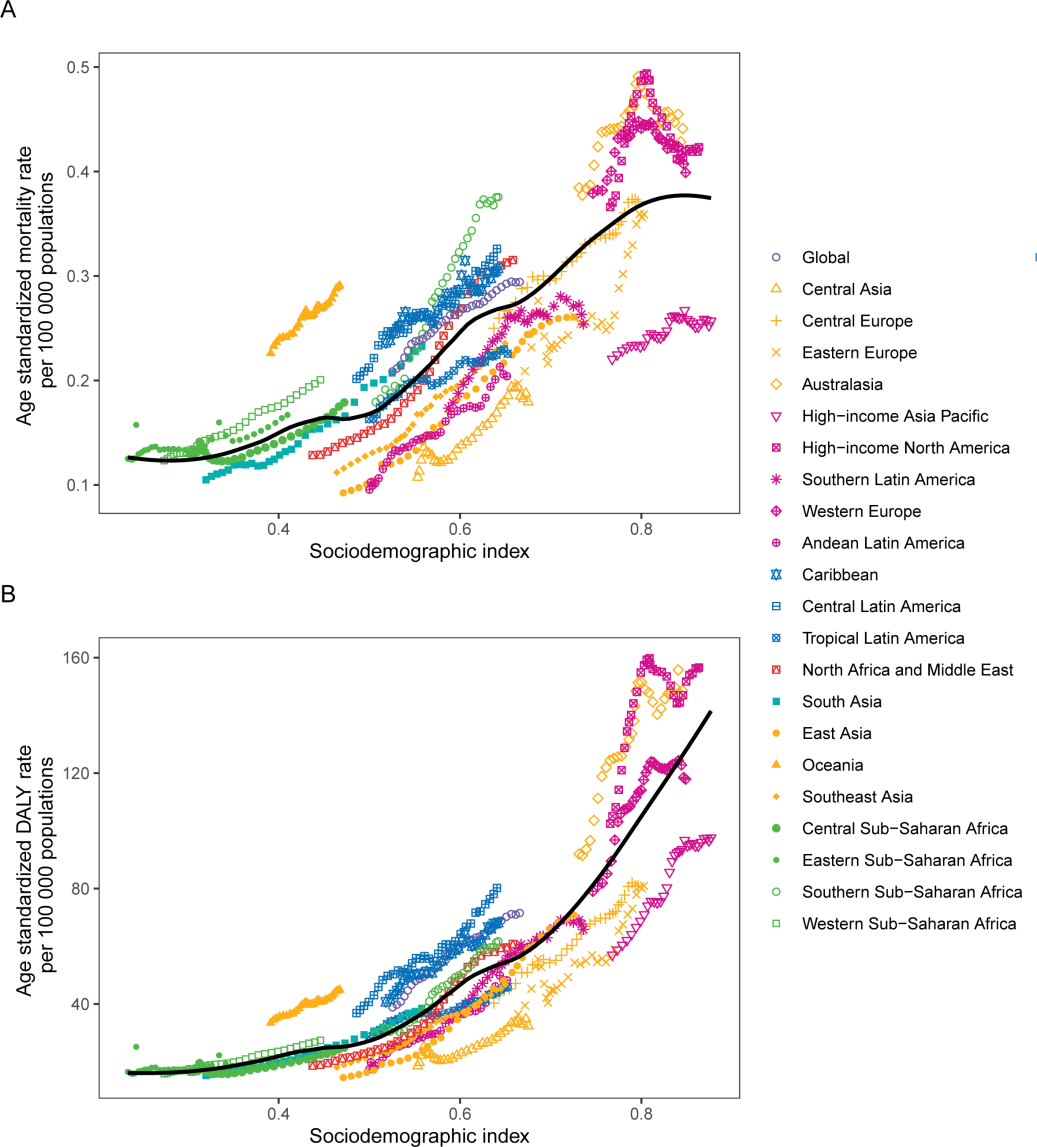
The relationship between the age-standardized mortality rates **(A)** and age standardized disability-adjusted life years (DALYs) rates **(B)** of bone and joint infections and the socio-demographic index (SDI) across global and 21 GBD regions. The solid line represents expected values based on the SDI level and mortality rate of each region. Each point represents the age-standardized rate and its corresponding SDI value for a specific region in a given year, with 32 data points per region illustrating trends from 1990 to 2021. Data points above the solid line indicate a higher-than-expected disease burden in that region, while those below the line indicate a lower-than-expected burden.

**Figure 3:**
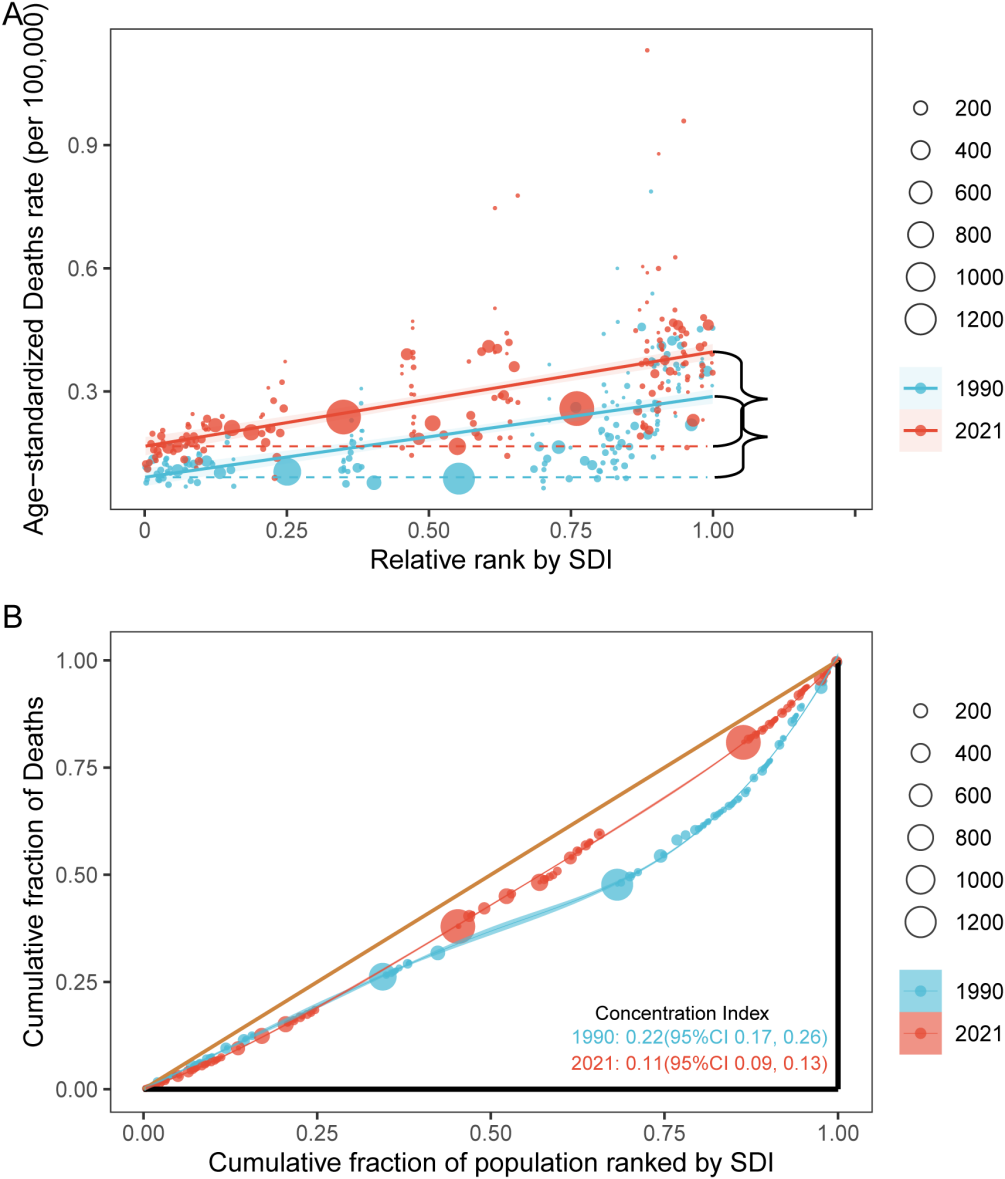
Health inequality regression curves **(A)** and concentration curves **(B)** for the mortality burden of bone and joint infections worldwide, 1990 and 2021.

Both the ASMR and the ASDR for BJIs showed a significant positive correlation with the SDI, a relationship further confirmed by health inequality analysis (**Figure 2**). Specifically, the slope index of inequality increased from 0.20 in 1990 to 0.23 in 2021, while the concentration index decreased from 0.22 to 0.11, suggesting that the overall disease burden of BJIs may have risen, leading to an increase in absolute inequality; however, its distribution across socioeconomic groups has become relatively more equitable (**Figure 3**).

### 3.6 Burden of BJIs by pathogens

From 1990 to 2021, the mortality and DALYs burden of BJIs caused by different pathogens worldwide showed a significant increasing trend, though the magnitude of change varied among pathogens. In terms of mortality burden, nearly all listed pathogens had higher numbers of deaths and ASMRs in 2021 compared to 1990. Among them, *Staphylococcus aureus* remained the leading pathogen, with 4,993 deaths and an ASMR of 0.06 per 100,000 in 2021, both substantially higher than other pathogens. Among Gram-negative bacteria, *Pseudomonas aeruginosa* and *Escherichia coli* contributed the heaviest mortality burden. From a long-term trend perspective, the ASMRs associated with most pathogens showed a significant increasing trend and *Enterococcus faecalis* and *Enterococcus faecium* exhibited the fastest growth rates. Notably, *Streptococcus pneumoniae* was the only pathogen whose ASMR showed a declining trend. A similar increasing pattern was observed in the overall disease burden measured by DALYs (**Supplementary Figures 4**, **S5**; **Supplementary Table 3**).

### 3.7 Burden of BJIs by antimicrobial resistance

**Figure 4:**
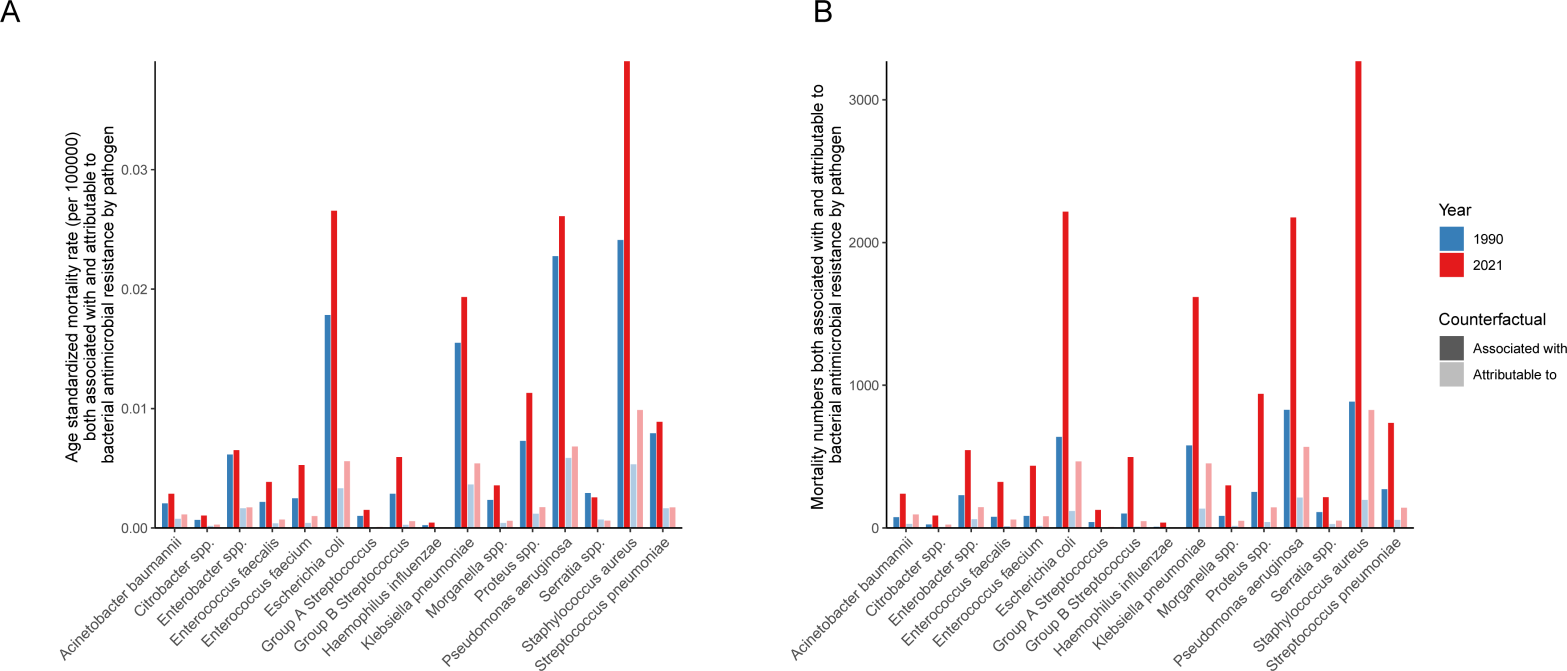
Bone and joint infections deaths attributable to and associated with antimicrobial resistance from different pathogens in 1990 and 2021. **(A)** Age standardized mortality rate (per 100000) with antimicrobial resistance; **(B)** Total mortality number with antimicrobial resistance.

To precisely quantify the independent impact of antimicrobial resistance, this study established two counterfactual scenarios: antimicrobial resistance-associated burden and antimicrobial resistance-attributable burden (GBD 2021 Antimicrobial Resistance Collaborators, 2024). In 2021, among BJIs, pathogens with higher numbers of deaths associated with antimicrobial resistance included *Staphylococcus aureus* (3,270 cases), *Escherichia coli* (2,216 cases), *Pseudomonas aeruginosa* (2,175 cases), and *Klebsiella pneumoniae* (1,618 cases). Among these, the numbers of deaths attributable to antimicrobial resistance were approximately 826 for *Staphylococcus aureus*, 568 for *Pseudomonas aeruginosa*, 466 for *Escherichia coli*, and 452 for *Klebsiella pneumoniae*. In contrast, the corresponding numbers of deaths associated with and attributable to antimicrobial resistance for these pathogens in 1990 were significantly lower. Furthermore, in terms of ASMRs, the antimicrobial resistance-associated mortality rates for *Staphylococcus aureus*, *Escherichia coli*, *Pseudomonas aeruginosa*, and *Klebsiella pneumoniae* in 2021 were 0.0391, 0.0266, 0.0261, and 0.0193 per 100,000 population, respectively. These rates increased from 0.0241, 0.0178, 0.0228, and 0.0155 in 1990. A similar increasing trend was observed for their attributable mortality rates. These findings indicate that over the past three decades, the burden of mortality related to antimicrobial resistance in major Gram-positive and Gram-negative pathogens has increased significantly in global bone and joint infection-related deaths (**Figure 4**).

It is important to note that these two metrics—antimicrobial resistance-associated and antimicrobial resistance-attributable burden—capture fundamentally different dimensions of the resistance problem. The antimicrobial resistance-associated burden represents the total health loss (deaths or DALYs) involving infections caused by drug-resistant pathogens; in counterfactual terms, it estimates the burden that could have been averted had no resistant infections occurred. By contrast, the antimicrobial resistance-attributable burden quantifies the *excess* health loss specifically due to resistance—the additional deaths or DALYs that arose because these infections were resistant rather than susceptible. For instance, among BJIs caused by *Staphylococcus aureus* in 2021, 3,270 deaths were associated with antimicrobial resistance, meaning they involved drug-resistant strains, while 826 deaths were attributable to antimicrobial resistance, representing the portion directly attributable to resistance per se. The difference between these two figures (2,444 deaths) reflects the burden that, while involving resistant organisms, would likely have occurred even with susceptible strains due to the inherent virulence of the pathogen and patient-level risk factors. This distinction is clinically meaningful: the associated burden informs the overall infection prevention and control agenda, highlighting the total disease toll linked to resistant pathogens, whereas the attributable burden more directly reflects the incremental harm imposed by resistance, providing a targeted metric for evaluating antimicrobial stewardship interventions, prioritizing investment in novel antibiotic development, and assessing the potential benefit of resistance containment strategies.

## 4 Discussion

This study presented the first systematic assessment of the disease burden, temporal trends, pathogen spectrum, and antimicrobial resistance profiles of BJIs at the global, regional, and national levels from 1990 to 2021, which demonstrated that during this period, deaths and DALYs due to BJIs increased substantially, with age-standardized rates showing a persistent upward trend. This growing burden was observed worldwide but exhibited significant geographical inequalities, with the most rapid increase occurring in East Asia. From an etiological perspective, *Staphylococcus aureus* remained the leading pathogen for mortality and health loss. More importantly, antimicrobial resistance emerged as a key driver of additional health loss, particularly in infections caused by pathogens such as *Staphylococcus aureus*, *Escherichia coli*, and *Klebsiella pneumoniae*.

Our findings carry three actionable messages for clinical practice. First, given the marked regional heterogeneity in pathogen distribution and resistance profiles, empirical antibiotic selection for BJIs should be informed by local surveillance data rather than relying on generalized international guidelines. Second, the growing antimicrobial resistance-attributable burden underscores the need to embed antimicrobial stewardship principles into orthopedic infection pathways, including routine de-escalation protocols and resistance-aware surgical prophylaxis. Third, the disproportionate burden in elderly populations highlights the importance of integrating geriatric and infectious disease expertise in the multidisciplinary management of BJIs.

The rise in mortality from BJIs is primarily driven by the combined effects of demographic shifts and evolving clinical practices. On one hand, population aging is one of the drivers for the rising mortality associated with BJIs. Older adults often experience declined immune function and have comorbidities such as diabetes and cardiovascular diseases, which not only elevate infection risk but also lead to poorer prognoses (Hu et al., 2025). Studies have found that the osteomyelitis mortality rate is highest among individuals aged 85 years and older (33.6 per 100,000 population), with the most pronounced increase observed in this age group (Braun et al., 2025). On the other hand, changes in clinical practice have also contributed to rising mortality. The continuous global increase in the volume of joint replacement surgeries has directly led to a growing number of periprosthetic joint infection cases. Currently, prosthetic joint infections account for over 40% of all bone and joint infection cases, making them the predominant clinical type (Sousa and Abreu, 2024). Furthermore, the spread of drug-resistant pathogens, such as methicillin-resistant *Staphylococcus aureus*, has reduced the effectiveness of antimicrobial therapy, while the presence of patient comorbidities may also limit treatment options and compromise outcomes (Hanlon et al., 2019). Additionally, the increased use of immunosuppressive therapies, including those for cancer chemotherapy and autoimmune diseases, may contribute to the heightened susceptibility to BJIs (Baden et al., 2024; Mansilla-Polo and Morgado-Carrasco, 2024).

Beyond demographic and pathogen-related factors, the role of surgical practices and perioperative infection control in shaping the BJIs burden deserves greater attention. The global expansion of orthopedic surgical volumes—including joint arthroplasty, spinal instrumentation, and trauma fixation—has substantially increased the population at risk for surgical site infections and periprosthetic infections (Li et al., 2026). Perioperative infection control measures, such as laminar airflow ventilation, antibiotic prophylaxis protocols, and skin decolonization strategies, vary widely across healthcare settings and geographic regions, and adherence to evidence-based bundles is often suboptimal, particularly in resource-limited environments (Sinha et al., 2014; Wong et al., 2019). Moreover, the increasing complexity of surgical procedures and the growing use of implantable devices create additional surfaces for biofilm formation, rendering infections more difficult to eradicate and more likely to recur (Semeshchenko et al., 2025). These factors collectively suggest that improvements in surgical infection prevention—including standardized perioperative protocols, enhanced operating room environments, and implant-specific infection control strategies—represent a critical yet underemphasized lever for reducing the global BJIs burden (Bratzler and Houck, 2004).

While the factors discussed above likely represent genuine drivers of increasing BJIs burden, it is equally important to acknowledge that the observed upward trends may, at least in part, reflect improvements in diagnostic capacity, changes in health information and reporting systems, and heightened clinical awareness rather than—or in addition to—a true increase in underlying disease incidence. These ascertainment-related factors warrant careful consideration to avoid overestimating the magnitude of epidemiological change. Several methodological and structural factors may account for the apparently rising incidence of BJIs. Population growth and demographic expansion have expanded the absolute population at risk, contributing to increases in the total number of BJIs cases even in the absence of changes in age-specific incidence rates (Kremers et al., 2015). Additionally, advances in diagnostic technologies—including molecular methods (16S rRNA sequencing, multiplex PCR, next-generation sequencing) and improved imaging modalities (high-resolution MRI, FDG-PET/CT)—have enhanced pathogen detection in previously culture-negative cases and enabled earlier identification of osteomyelitis and prosthetic joint infections, thereby increasing the proportion of cases captured clinically (Zhang et al., 2024; Li et al., 2026). Concurrently, substantial expansions in health information infrastructure—encompassing strengthened vital registration systems, the progressive adoption of ICD-10/ICD-11 coding, and the establishment of antimicrobial resistance surveillance networks such as WHO GLASS and EARS-Net—have improved the completeness and granularity of infection-related data, particularly in low- and middle-income countries (Cassini et al., 2019; Organization, 2022). Together with heightened clinical awareness, greater adherence to guidelines recommending routine microbiological sampling, and the integration of multidisciplinary teams in BJIs management, these developments have likely reduced the historical burden of undetected or misclassified cases, contributing to the observed upward trends in reported BJIs incidence and associated mortality (Hanssen et al., 2025). It should be noted that the GRAM framework, by relying on administrative and surveillance data, cannot fully disentangle the contributions of true epidemiological change from improvements in ascertainment. The increasing trends reported in this study should therefore be interpreted as reflecting the net effect of multiple drivers, including both genuine increases in disease burden—driven by aging, surgical volume expansion, and the spread of antimicrobial resistance—and improved detection and reporting. Future studies employing primary data collection with standardized diagnostic criteria across time periods would be valuable in estimating the relative contributions of these factors to the observed trends.

While the global total number of deaths from BJIs has risen significantly, its distribution exhibits marked geographic disparities. East Asia bears the heaviest mortality burden, demonstrating the most rapid increase, a trend likely driven by multifactorial causes. First, population aging is a key risk factor. For instance, the mean age of patients with spinal osteomyelitis in Japan is about 69 years, and the most common age group for infectious spondylitis in South Korea is 60–79 years (Akiyama et al., 2013; Park, 2024). Second, the epidemics of diabetes and obesity have exacerbated the disease burden. In East Asia, approximately 45.7% of type 2 diabetes-related DALYs are attributable to high body mass index (GBD 2021 Diabetes Collaborators, 2023). Furthermore, the region has seen one of the world’s largest increases in the ASDR for osteoarthritis due to high body mass index (Yang et al., 2024). Concurrently, the volume of joint replacement surgeries has surged in East Asia, particularly in China. Periprosthetic joint infection—a major complication following arthroplasty—significantly increases mortality risk. Studies indicate that the one-year mortality rate for patients undergoing revision surgery due to infection can reach 10.6%, approximately five times higher than that for revisions performed for aseptic failure (Zmistowski et al., 2013; Shakya et al., 2024). Additionally, antimicrobial resistance poses a serious challenge. The number of deaths attributable to BJIs caused by fluoroquinolone-resistant *Escherichia coli* has shown a marked upward trend, making it one of the fastest-growing infection syndrome–pathogen–drug combinations (X, 2025).

This study found that Bahrain is among the countries with the highest mortality burden from BJIs. On the one hand, the spread of drug-resistant bacteria, such as methicillin-resistant *Staphylococcus aureus* (MRSA), poses a significant threat. The detection rate of MRSA in governmental healthcare institutions is approximately 22%, with most cases being community-acquired, which substantially increases the risk of skin and soft tissue infections progressing to osteomyelitis or septic arthritis (Khawaja et al., 2021). On the other hand, up to one-third of the population in the region is overweight, and approximately 42.8% are obese, while the prevalence of diabetes ranks among the highest globally, which significantly elevate the risk of secondary osteomyelitis (Alawainati et al., 2023). Furthermore, although an infection prevention and control framework exists in the region, there is still room for optimization in practical implementation, training support, and resource allocation (Sowar et al., 2024). Studies suggest that antimicrobial stewardship programs remain in early stages of development, with limited integration into surgical pathways and inconsistent adherence to perioperative prophylaxis guidelines, which may compromise infection control effectiveness in orthopedic settings (Alghamdi, 2018; Sowar et al., 2024). Additionally, the high volume of elective orthopedic procedures performed in the region, combined with the strain on healthcare systems posed by the dual burden of chronic diseases and surgical demand, may further elevate the risk of healthcare-associated BJIs (GBD 2015 Eastern Mediterranean Region Diabetes and Chronic Kidney Disease Collaborators, 2018).

Regions with higher SDI are associated with a greater mortality burden from BJIs, which may reflect systemic gaps in healthcare access, infection control, and antimicrobial stewardship. In Africa, only about 17% of the population is covered by health insurance, with most patients experiencing delayed healthcare-seeking and limited microbiological diagnostic capacity (Organization, 2020). Data show that in sub-Saharan Africa, resistance of *Escherichia coli* to third-generation cephalosporins ranges from 0% to 87%, and methicillin resistance in *Staphylococcus aureus* ranges from 0% to 100%—both of which are key pathogens in BJIs (Essack et al., 2017). In Europe, approximately 75% of the antimicrobial resistance burden is healthcare-associated (Cassini et al., 2019). Although infection prevention and control programs can significantly reduce antimicrobial resistance and associated mortality, as of 2022, only some countries have fully implemented them (Organization, 2023). In South Asia, while all countries have developed national action plans on antimicrobial resistance, weak regulatory authorities and the impact of the COVID-19 pandemic have hindered the effective implementation of multiple interventions (Sihombing et al., 2023). In contrast, in East Asia, China has made notable progress in hospital antimicrobial stewardship (Chen et al., 2023). Disparities across regions in mitigating antimicrobial resistance continue to influence the distribution of pathogens causing bone and joint infections and shape the evolving resistance trends. These geographical disparities carry direct implications for clinical management: in high-burden regions such as East Asia and the Gulf States, empirical antibiotic regimens for suspected BJIs should account for the locally prevalent resistance profiles—for instance, the high MRSA prevalence in Bahrain warrants broader first-line coverage compared to regions where methicillin-susceptible (MSSA) predominates. Locally adapted clinical guidelines, informed by routine antimicrobial surveillance data, are therefore essential rather than the uniform application of international protocols (Pulcini and Gyssens, 2013).

The composition and distribution of causative pathogens for BJIs exhibit significant geographical heterogeneity. While *Staphylococcus aureus* remains the most common pathogen globally, the proportion of MSSA versus MRSA strains varies drastically across different regions. For instance, the prevalence of MSSA infection can exceed 90% in Finland, compared to 11%-61% in France. The proportion of MRSA infections can be as high as 40%-50% in parts of the United States, yet is relatively low or rare in the United Kingdom, Spain, and Nordic countries (Saavedra-Lozano et al., 2017). Furthermore, coagulase-negative *staphylococci* and *streptococci* may surpass *Staphylococcus aureus* as the most prevalent pathogens in prosthetic joint infections in certain European countries (Phillips et al., 2006; Stefánsdóttir et al., 2009). The proportion of Gram-negative bacteria also shows regional variation, ranging from 5% to 23% (Zmistowski et al., 2011). Notably, geographical differences in the pathogen spectrum of BJIs in children are also pronounced. For example, a Swiss study revealed that *Kingella kingae* (47.8%) was the most common pathogen in pediatric BJIs, exceeding *Staphylococcus aureus* (35.5%) (Juchler et al., 2018). These geographical disparities in pathogen distribution have direct implications for guiding the selection of empirical antibiotic therapy. From a clinical practice perspective, these findings highlight that orthopedic and infectious disease teams should integrate local microbiological data into empirical treatment pathways and consider routine preoperative or intraoperative culture sampling protocols tailored to the pathogen ecology of their specific setting.

A core finding of our study is that the global burden of BJIs linked to antimicrobial resistance is increasing persistently across both associated and attributable metrics, which is consistent with previous research (Lewandrowski et al., 2024). This dual increase is clinically significant: the rising antimicrobial resistance-associated burden indicates that resistant organisms are playing an expanding role in the overall BJI disease landscape, reflecting broader trends in pathogen ecology and healthcare-associated transmission, while the concurrent rise in antimicrobial resistance-attributable burden demonstrates that resistance is generating an increasing excess toll beyond what would be expected from susceptible infections alone. The latter finding carries direct implications for antimicrobial stewardship, as it suggests that current therapeutic strategies are becoming progressively less effective at mitigating the adverse consequences of resistant infections. Key resistant pathogens, such as MRSA, *Streptococcus*, and *Escherichia coli*, have shown rapidly rising resistance rates, and the gap between their associated and attributable burden underscores that while these organisms cause substantial harm regardless of resistance phenotype, the incremental harm attributable to resistance represents a growing and modifiable component of the total burden (Lewandrowski et al., 2024). For instance, the resistance rate to erythromycin in joint infections reached as high as 30.81%, marking an increase of approximately 20% over the past decade (2022). Furthermore, the distribution of this burden exhibits significant geographical disparities, which are closely associated with socioeconomic development levels. Regions with limited access to healthcare resources and weak antimicrobial stewardship are facing a more severe impact (Lewandrowski et al., 2024). These findings underscore the urgent need for multi-faceted interventions, including stringent infection prevention measures, strengthened rational use of antibiotics informed by local resistance surveillance data, and accelerated development of novel therapeutic and diagnostic approaches.

For antimicrobial stewardship programs, our findings provide a quantitative basis for prioritization: the persistently widening gap between antimicrobial resistance-associated and antimicrobial resistance-attributable burden for key pathogens such as *Staphylococcus aureus* suggests that stewardship interventions targeting these organism–syndrome combinations could yield the greatest absolute reductions in preventable mortality. Embedding syndrome-specific resistance surveillance into orthopedic infection pathways—particularly for prosthetic joint infections and postoperative osteomyelitis—represents a practical strategy for translating population-level resistance trends into bedside clinical decision-making.

This study has several limitations. First, input data coverage is geographically uneven, with denser representation from high-income countries; estimates for data-sparse regions rely more heavily on covariate-based extrapolation and thus carry greater uncertainty. Second, the mapping of ICD-coded diagnoses to infectious syndromes involves assumptions about the consistency of coding practices across heterogeneous healthcare systems. Third, the relative risk estimates used to compute attributable antimicrobial resistance burden are derived from observational studies and may be subject to residual confounding. Fourth, the reported 95% UIs capture between-iteration parameter uncertainty but may not fully incorporate all sources of uncertainty, including systematic biases in input data and unmeasured spatial heterogeneity. Furthermore, this study estimated BJIs as a broad category and did not provide a detailed breakdown of the burden by specific anatomical sites (e.g., knee vs. hip infections, spinal infections). Given that infections at different sites may vary in etiology, risk factors, treatment pathways, and functional outcomes, this aggregated estimation framework limits a deeper understanding of the heterogeneity in disease burden and hinders the development of targeted prevention and resource allocation strategies for specific anatomical locations. Therefore, future studies should pursue more anatomically resolved burden estimates, where data permit, to address this knowledge gap. Additionally, the temporal trends reported in this study may partly reflect improved case ascertainment rather than true epidemiological change. Over the 31-year study period, population growth has expanded the at-risk population, while advances in diagnostic technologies, broader access to microbiological and imaging services, and evolving case definitions have enhanced detection and reporting completeness, particularly in low- and middle-income countries. Although age-standardized rates mitigate the influence of demographic shifts, the administrative data underlying the GRAM framework cannot fully distinguish genuine increases in disease burden from improvements in surveillance capacity. Finally, this study focused solely on bacterial pathogens and did not include other pathogens such as fungi.

## 5 Conclusion

This study provides the first systematic assessment of the global, regional, and national burden of BJIs, including their temporal trends, pathogen spectrum, and antimicrobial resistance profiles, from 1990 to 2021. The results indicate that during this period, deaths and DALYs attributable to BJIs increased substantially, with a persistent upward trend in age-standardized rates, which was observed globally but was marked by significant geographical disparities, with the fastest growth occurring in East Asia. From an etiological perspective, *Staphylococcus aureus* remained the leading pathogen responsible for mortality and health loss. More importantly, antimicrobial resistance has emerged as a key driver of additional health loss, particularly in infections caused by pathogens such as *Staphylococcus aureus*, *Escherichia coli*, and *Klebsiella pneumoniae*. This study offers crucial scientific evidence for understanding the evolving epidemiology of BJIs, identifying high-risk regions and populations, and informing targeted strategies for infection prevention, control, and antimicrobial stewardship. Future efforts should focus on conducting more refined burden studies based on enhanced surveillance systems to ultimately improve the overall prevention and management of BJIs globally.

## Data Availability

Data sources are accessible through the Global Research on Antimicrobial Resistance (GRAM) project.

## Supplementary material

Supplementary Figure 1 The trends in numbers and age standardized mortality rates **(A)** and age standardized disability-adjusted life years (DALYs) rates **(C)** for bone and joint infections burden from 1990 to 2021. Age-specific numbers and rates of deaths **(B)** and disability-adjusted life years (DALYs) **(D)** for bone and joint infections burden by sex in 2021. Error bars indicate the 95% uncertainty interval for numbers. Shading indicates the 95% uncertainty interval for rates.

Supplementary Figure 2 The numbers and rates of deaths **(A)** and disability-adjusted life years (DALYs) **(B)** for bone and joint infections across 21 GBD regions in 2021. Error bars indicate the 95% uncertainty interval for numbers. Shading indicates the 95% uncertainty interval for rates.

Supplementary Figure 3 The global map of age standardized mortality rates **(A)**, estimated annual percent change of age standardized mortality rates **(B)**, age standardized disability-adjusted life years (DALYs) rates **(C)** and estimated annual percent change of age standardized mortality rates **(D)** of bone and joint infections in 204 countries and territories.

Supplementary Figure 4 Age standardized mortality rates and total mortality numbers of different pathogens in bone and joint infections from 1990 to 2021.

Supplementary Figure 5 Heatmap ranking of mortality attributable to various pathogens causing bone and joint infections from 1990 to 2021.
