# Supplementary figures and images for: Global, regional, and national burden of bone and joint infections, 1990–2021: a comprehensive analysis of trends, pathogens, and antimicrobial resistance
Source: Front Cell Infect Microbiol. 2026 Jun 2;16:1858745. doi: 10.3389/fcimb.2026.1858745 (PMC13269380; doi:10.3389/fcimb.2026.1858745)

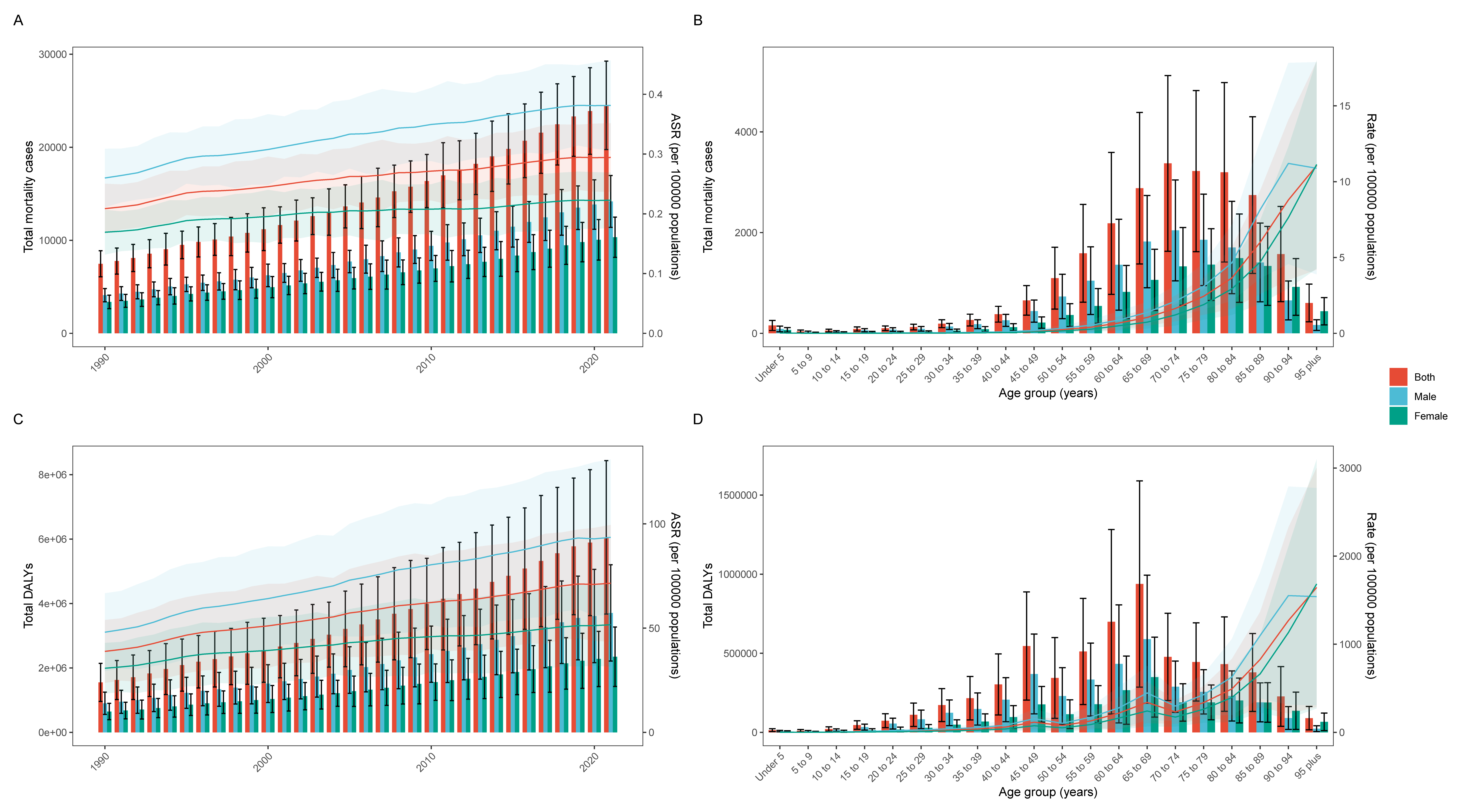

Supplement: Supplementary Figure 1 — The trends in numbers and age standardized mortality rates (A) and age standardized disability-adjusted life years (DALYs) rates (C) for bone and joint infections burden from 1990 to 2021. Age-specific numbers and rates of deaths (B) and disability-adjusted life years (DALYs) (D) for bone and joint infections burden by sex in 2021. Error bars indicate the 95% uncertainty interval for numbers. Shading indicates the 95% uncertainty interval for rates. [file Image1.tif]

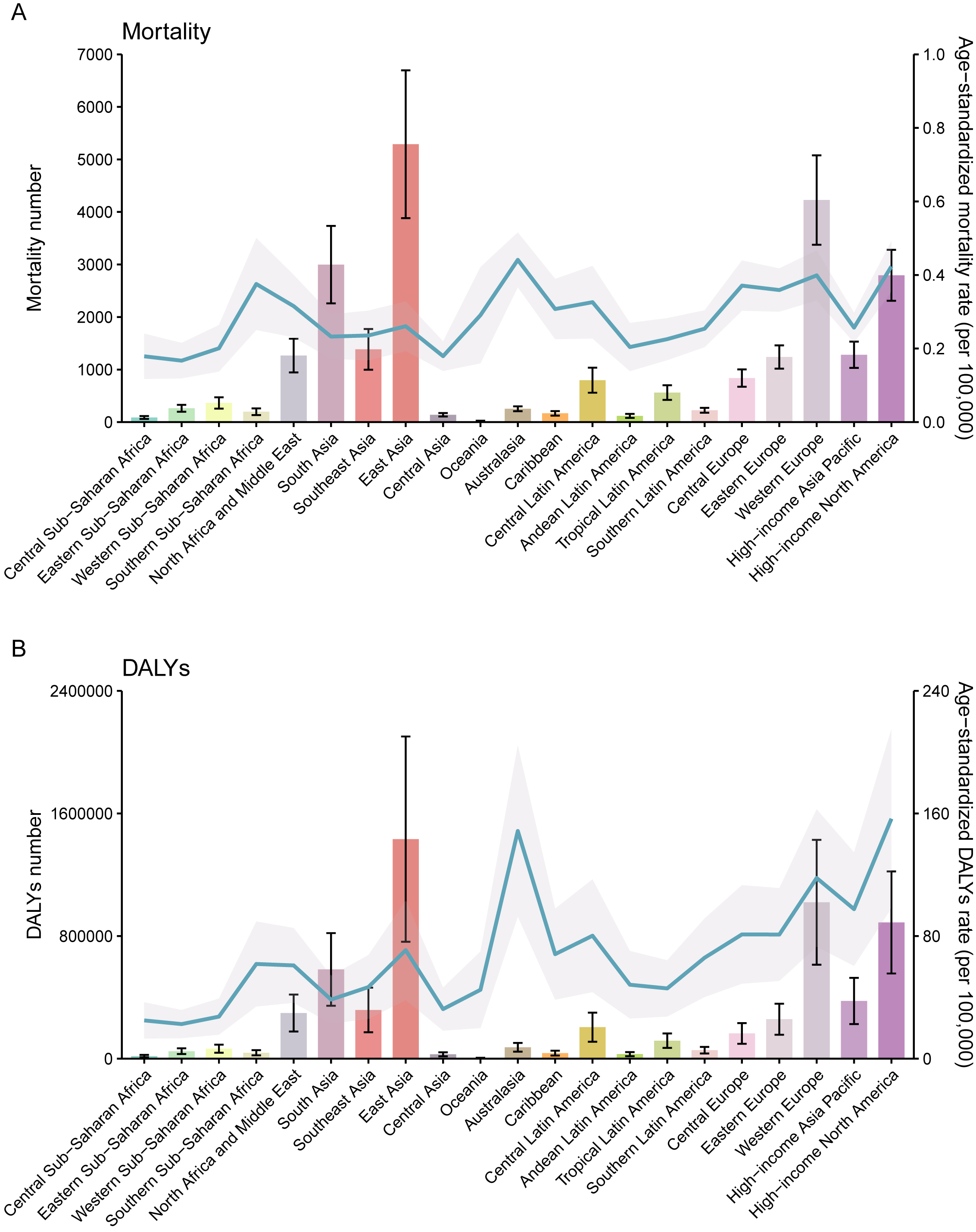

Supplement: Supplementary Figure 2 — The numbers and rates of deaths (A) and disability-adjusted life years (DALYs) (B) for bone and joint infections across 21 GBD regions in 2021. Error bars indicate the 95% uncertainty interval for numbers. Shading indicates the 95% uncertainty interval for rates. [file Image2.tif]

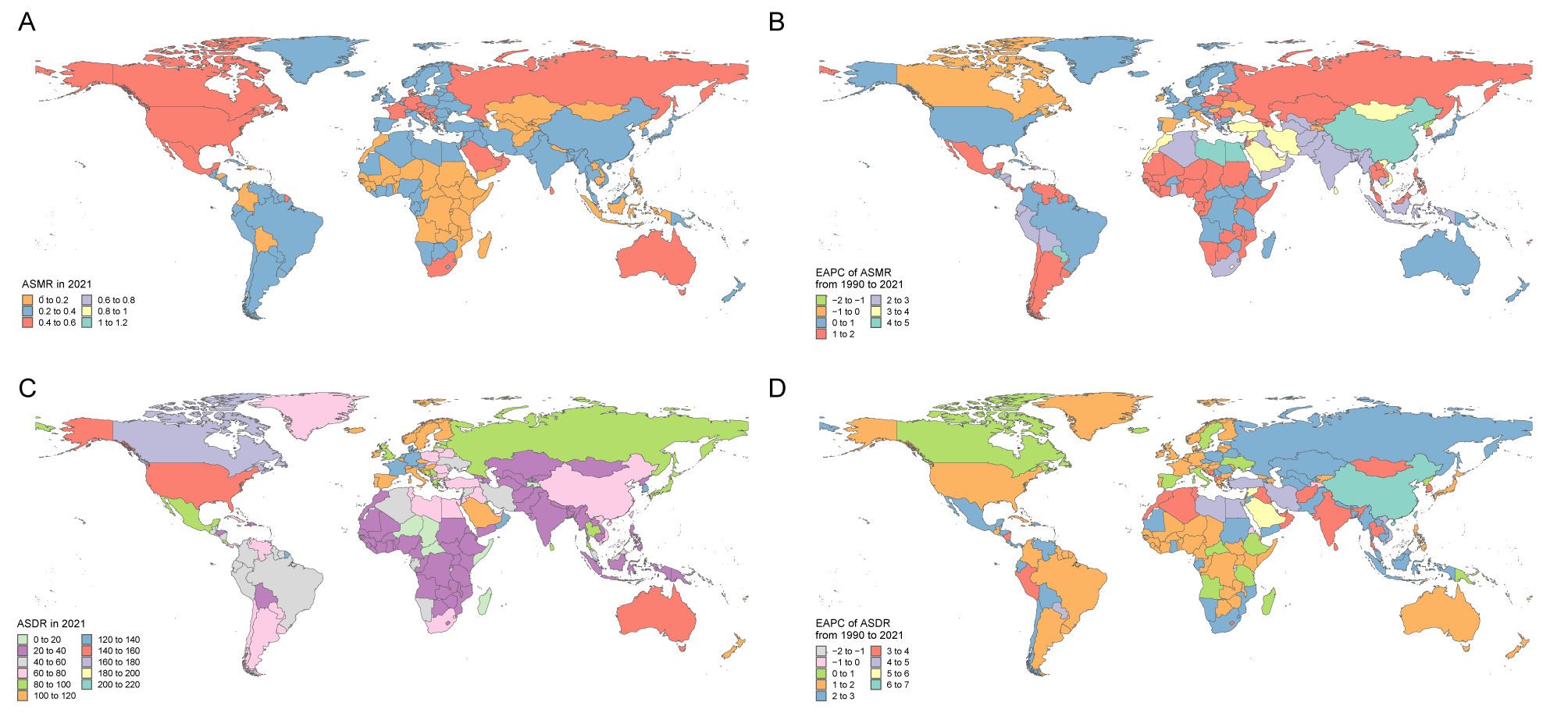

Supplement: Supplementary Figure 3 — The global map of age standardized mortality rates (A), estimated annual percent change of age standardized mortality rates (B), age standardized disability-adjusted life years (DALYs) rates (C) and estimated annual percent change of age standardized mortality rates (D) of bone and joint infections in 204 countries and territories. [file Image3.tif]

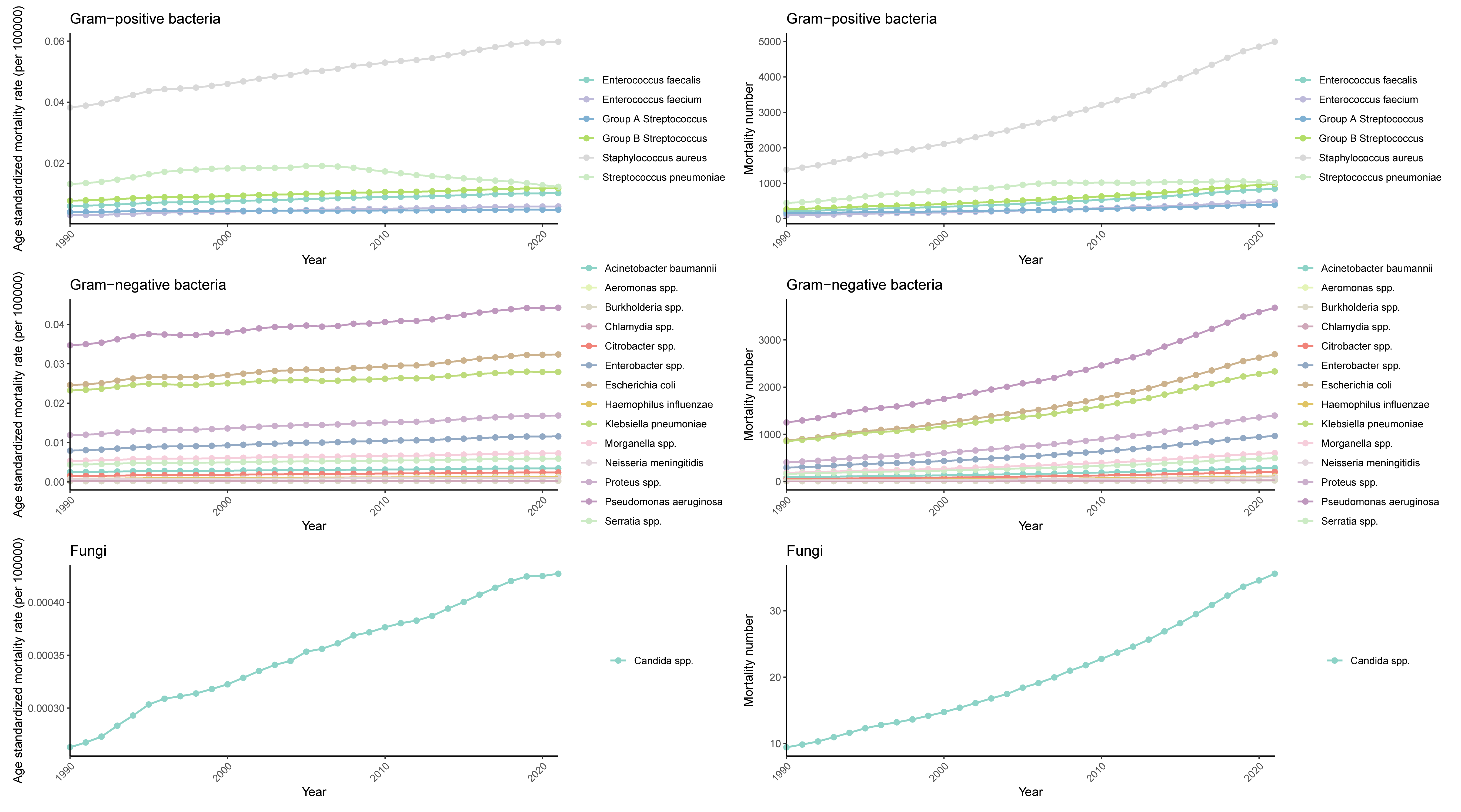

Supplement: Supplementary Figure 4 — Age standardized mortality rates and total mortality numbers of different pathogens in bone and joint infections from 1990 to 2021. [file Image4.tif]

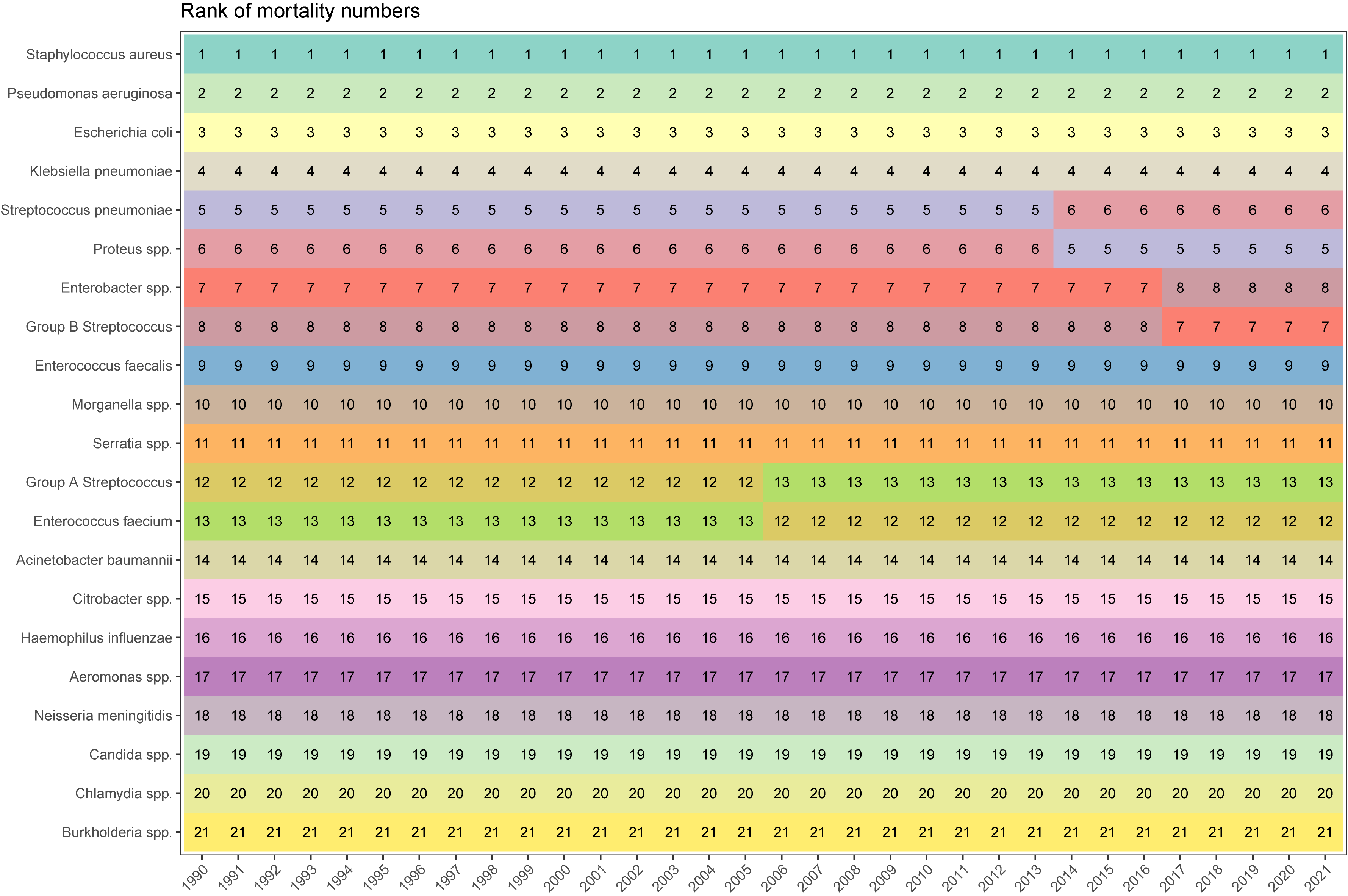

Supplement: Supplementary Figure 5 — Heatmap ranking of mortality attributable to various pathogens causing bone and joint infections from 1990 to 2021. [file Image5.tif]
