# Supplementary material for: Global, regional, and national burden of bone and joint infections, 1990–2021: a comprehensive analysis of trends, pathogens, and antimicrobial resistance
Source: Front Cell Infect Microbiol. 2026 Jun 2;16:1858745. doi: 10.3389/fcimb.2026.1858745 (PMC13269380; doi:10.3389/fcimb.2026.1858745)
Supplement: Supplementary file 6 [file Table1.docx]

Table S1 Osteoarticular infections mortality and DALYs in 1990 and 2021 for both sexes and estimated annual percentage change in age-standardized rates by location

|  | Deaths | | | | | DALYs | | | | |
| --- | --- | --- | --- | --- | --- | --- | --- | --- | --- | --- |
| Location | All ages number in 1990 (95% UI) | All ages number in 2021 (95% UI) | Age-standardized rate in 1990 (95% UI) | Age-standardized rate in 2021 (95% UI) | Estimated annual percentage change (95% CI),1990-2021 | All ages number in 1990 (95% UI) | All ages number in 2021 (95% UI) | Age-standardized rate in 1990 (95% UI) | Age-standardized rate in 2021 (95% UI) | Estimated annual percentage change (95% CI),1990-2021 |
| Global | 7475 (6076, 8874) | 24505 (19756, 29253) | 0.21 (0.17, 0.25) | 0.29 (0.24, 0.35) | 1.06 (0.99, 1.13) | 1549957 (959970, 2139944) | 6054611 (3674488, 8434734) | 38.84 (23.76, 53.92) | 71.53 (43.56, 99.51) | 1.94 (1.82, 2.05) |
| GBD regions |  |  |  |  |  |  |  |  |  |  |
| Central Sub-Saharan Africa | 31 (17, 44) | 87 (59, 115) | 0.13 (0.08, 0.17) | 0.18 (0.12, 0.24) | 0.88 (0.60, 1.16) | 4908 (2668, 7149) | 16749 (8986, 24512) | 16.53 (9.03, 24.04) | 24.94 (13.12, 36.76) | 1.11 (0.76, 1.46) |
| Eastern Sub-Saharan Africa | 109 (65, 153) | 263 (197, 329) | 0.13 (0.09, 0.16) | 0.17 (0.12, 0.22) | 0.71 (0.52, 0.91) | 18544 (10280, 26808) | 48431 (29795, 67067) | 16.52 (9.73, 23.31) | 22.55 (13.34, 31.77) | 0.75 (0.42, 1.09) |
| Western Sub-Saharan Africa | 113 (79, 147) | 365 (256, 473) | 0.12 (0.09, 0.16) | 0.20 (0.14, 0.26) | 1.62 (1.54, 1.70) | 17501 (10586, 24416) | 64956 (38419, 91494) | 15.88 (9.06, 22.70) | 27.43 (15.67, 39.20) | 1.79 (1.69, 1.89) |
| Southern Sub-Saharan Africa | 50 (35, 66) | 197 (134, 260) | 0.18 (0.12, 0.24) | 0.38 (0.25, 0.50) | 2.70 (2.52, 2.88) | 9914 (5499, 14330) | 38235 (21364, 55106) | 29.56 (15.91, 43.20) | 61.77 (34.04, 89.50) | 2.71 (2.56, 2.87) |
| North Africa and Middle East | 214 (170, 258) | 1266 (946, 1586) | 0.13 (0.10, 0.16) | 0.32 (0.23, 0.40) | 3.39 (3.23, 3.55) | 38969 (24735, 53203) | 297385 (176991, 417780) | 18.68 (11.36, 26.00) | 60.85 (36.43, 85.26) | 4.42 (4.22, 4.62) |
| South Asia | 570 (380, 760) | 2997 (2260, 3734) | 0.10 (0.07, 0.14) | 0.23 (0.17, 0.29) | 2.65 (2.43, 2.86) | 104123 (60245, 148001) | 582163 (345024, 819302) | 15.20 (8.58, 21.82) | 38.54 (22.96, 54.12) | 3.11 (2.91, 3.30) |
| Southeast Asia | 268 (198, 338) | 1385 (997, 1772) | 0.11 (0.08, 0.14) | 0.24 (0.17, 0.30) | 2.41 (2.35, 2.47) | 55557 (33079, 78034) | 317564 (171723, 463405) | 18.07 (10.42, 25.72) | 46.69 (25.82, 67.56) | 3.04 (2.96, 3.12) |
| East Asia | 694 (547, 842) | 5289 (3883, 6695) | 0.09 (0.07, 0.11) | 0.26 (0.19, 0.33) | 3.74 (3.50, 3.98) | 136442 (83463, 189420) | 1432594 (762894, 2102293) | 14.51 (8.80, 20.21) | 70.79 (38.31, 103.27) | 5.81 (5.57, 6.06) |
| Central Asia | 52 (42, 61) | 139 (106, 172) | 0.11 (0.09, 0.13) | 0.18 (0.14, 0.22) | 1.85 (1.61, 2.09) | 9692 (5984, 13399) | 28425 (15662, 41187) | 18.51 (11.31, 25.72) | 32.34 (18.29, 46.39) | 1.89 (1.58, 2.21) |
| Oceania | 6 (3, 8) | 18 (10, 26) | 0.23 (0.11, 0.34) | 0.29 (0.16, 0.42) | 0.66 (0.59, 0.73) | 1120 (465, 1776) | 3702 (1643, 5761) | 33.47 (13.10, 53.84) | 44.93 (19.96, 69.90) | 0.77 (0.69, 0.86) |
| Australasia | 88 (73, 104) | 253 (206, 301) | 0.38 (0.32, 0.45) | 0.44 (0.37, 0.52) | 0.45 (0.24, 0.65) | 21092 (12957, 29228) | 73914 (45317, 102510) | 91.97 (56.56, 127.38) | 148.56 (92.73, 204.39) | 1.54 (1.25, 1.82) |
| Caribbean | 58 (42, 75) | 167 (123, 210) | 0.24 (0.17, 0.30) | 0.31 (0.23, 0.39) | 0.78 (0.67, 0.90) | 10905 (6224, 15586) | 36248 (20481, 52015) | 40.77 (22.77, 58.76) | 68.20 (38.51, 97.88) | 1.58 (1.45, 1.72) |
| Central Latin America | 166 (123, 209) | 798 (559, 1037) | 0.20 (0.14, 0.26) | 0.33 (0.23, 0.42) | 1.34 (1.22, 1.47) | 36334 (21410, 51257) | 205375 (110634, 300116) | 36.85 (20.75, 52.96) | 80.22 (43.43, 117.01) | 2.24 (2.08, 2.39) |
| Andean Latin America | 21 (16, 26) | 118 (81, 156) | 0.10 (0.07, 0.12) | 0.20 (0.14, 0.27) | 2.42 (2.26, 2.58) | 4461 (2719, 6202) | 29621 (16123, 43120) | 17.45 (10.48, 24.42) | 48.22 (26.19, 70.26) | 3.25 (3.09, 3.41) |
| Tropical Latin America | 142 (107, 177) | 561 (422, 701) | 0.16 (0.12, 0.21) | 0.23 (0.17, 0.28) | 1.05 (0.95, 1.14) | 30363 (17899, 42827) | 116947 (69909, 163984) | 28.62 (16.74, 40.49) | 45.84 (27.49, 64.19) | 1.35 (1.23, 1.47) |
| Southern Latin America | 77 (61, 93) | 225 (179, 271) | 0.17 (0.14, 0.21) | 0.25 (0.20, 0.30) | 1.23 (0.97, 1.49) | 15926 (9497, 22355) | 55221 (33620, 76821) | 34.51 (20.62, 48.40) | 65.84 (40.24, 91.45) | 2.11 (1.85, 2.37) |
| Central Europe | 353 (288, 417) | 838 (673, 1003) | 0.25 (0.20, 0.30) | 0.37 (0.30, 0.44) | 1.17 (1.09, 1.25) | 57663 (34955, 80370) | 164443 (96976, 231910) | 40.30 (24.72, 55.89) | 81.07 (48.92, 113.22) | 2.17 (2.05, 2.30) |
| Eastern Europe | 518 (436, 601) | 1239 (1018, 1459) | 0.20 (0.16, 0.23) | 0.36 (0.30, 0.42) | 1.41 (1.05, 1.76) | 93906 (58544, 129268) | 257143 (156014, 358271) | 36.47 (22.80, 50.15) | 80.99 (50.73, 111.25) | 2.09 (1.68, 2.49) |
| Western Europe | 2226 (1768, 2684) | 4226 (3375, 5078) | 0.38 (0.31, 0.45) | 0.40 (0.33, 0.47) | 0.15 (-0.03, 0.33) | 427352 (257352, 597351) | 1020282 (612523, 1428041) | 79.04 (47.98, 110.10) | 117.89 (73.06, 162.72) | 1.27 (0.99, 1.55) |
| High-income Asia Pacific | 420 (348, 492) | 1282 (1031, 1532) | 0.22 (0.18, 0.26) | 0.26 (0.22, 0.30) | 0.46 (0.35, 0.57) | 112592 (69209, 155976) | 376539 (225713, 527366) | 57.04 (35.06, 79.02) | 97.58 (60.53, 134.63) | 1.80 (1.59, 2.01) |
| High-income North America | 1302 (1047, 1556) | 2795 (2310, 3280) | 0.37 (0.30, 0.43) | 0.42 (0.35, 0.49) | 0.09 (-0.22, 0.40) | 342748 (207790, 477706) | 888865 (555675, 1222055) | 102.46 (62.43, 142.50) | 156.56 (98.19, 214.93) | 1.00 (0.66, 1.34) |
| 204 countries and territories |  |  |  |  |  |  |  |  |  |  |
| Afghanistan | 6 (4, 8) | 26 (15, 36) | 0.09 (0.06, 0.11) | 0.19 (0.13, 0.25) | 2.73 (2.43, 3.04) | 971 (548, 1395) | 6345 (2448, 10241) | 11.99 (6.58, 17.40) | 31.75 (17.57, 45.93) | 3.13 (2.71, 3.56) |
| Albania | 2 (2, 3) | 8 (6, 10) | 0.11 (0.09, 0.13) | 0.19 (0.14, 0.24) | 2.04 (1.75, 2.33) | 350 (222, 478) | 1394 (761, 2027) | 15.17 (9.51, 20.82) | 35.83 (19.94, 51.73) | 3.31 (2.84, 3.77) |
| Algeria | 12 (9, 15) | 71 (49, 93) | 0.11 (0.08, 0.14) | 0.24 (0.16, 0.32) | 2.87 (2.75, 3.00) | 2334 (1336, 3332) | 16028 (9187, 22869) | 15.82 (8.91, 22.73) | 44.53 (25.24, 63.82) | 3.52 (3.41, 3.62) |
| American Samoa | 0 (0, 0) | 0 (0, 0) | 0.35 (0.20, 0.49) | 0.50 (0.30, 0.69) | 1.07 (0.77, 1.37) | 14 (7, 21) | 42 (20, 64) | 55.31 (25.76, 84.86) | 87.96 (44.42, 131.50) | 1.35 (1.03, 1.67) |
| Andorra | 0 (0, 0) | 0 (0, 1) | 0.32 (0.19, 0.45) | 0.30 (0.19, 0.40) | -0.01 (-0.26, 0.25) | 43 (19, 67) | 139 (63, 214) | 74.83 (32.96, 116.69) | 93.89 (42.91, 144.88) | 0.91 (0.57, 1.26) |
| Angola | 7 (2, 11) | 19 (13, 26) | 0.13 (0.08, 0.18) | 0.18 (0.12, 0.25) | 0.85 (0.55, 1.14) | 1092 (489, 1695) | 3706 (1800, 5612) | 17.43 (9.03, 25.83) | 25.36 (12.30, 38.42) | 0.98 (0.59, 1.37) |
| Antigua and Barbuda | 0 (0, 0) | 0 (0, 1) | 0.34 (0.22, 0.45) | 0.48 (0.35, 0.62) | 0.99 (0.80, 1.18) | 33 (17, 48) | 98 (55, 140) | 60.04 (31.84, 88.23) | 94.56 (53.53, 135.59) | 1.44 (1.28, 1.61) |
| Argentina | 54 (42, 66) | 143 (112, 174) | 0.17 (0.14, 0.21) | 0.25 (0.20, 0.31) | 1.13 (0.87, 1.40) | 10660 (6279, 15040) | 33544 (20251, 46837) | 33.54 (19.86, 47.22) | 61.82 (37.44, 86.21) | 1.91 (1.63, 2.19) |
| Armenia | 4 (3, 5) | 10 (7, 12) | 0.16 (0.12, 0.20) | 0.23 (0.18, 0.28) | 1.52 (1.06, 1.98) | 739 (431, 1047) | 1810 (978, 2642) | 25.79 (14.85, 36.73) | 44.04 (25.08, 63.01) | 2.46 (1.94, 2.98) |
| Australia | 75 (61, 88) | 219 (178, 260) | 0.39 (0.32, 0.46) | 0.45 (0.37, 0.53) | 0.48 (0.26, 0.69) | 18419 (11305, 25533) | 65591 (40205, 90977) | 96.38 (59.23, 133.53) | 156.98 (97.91, 216.04) | 1.56 (1.26, 1.87) |
| Austria | 52 (41, 63) | 89 (71, 107) | 0.43 (0.35, 0.51) | 0.44 (0.36, 0.52) | 0.52 (0.17, 0.87) | 10124 (5964, 14284) | 21625 (13131, 30119) | 90.92 (54.14, 127.70) | 127.34 (79.06, 175.63) | 1.36 (0.93, 1.80) |
| Azerbaijan | 5 (4, 5) | 14 (9, 18) | 0.09 (0.07, 0.11) | 0.14 (0.10, 0.19) | 1.78 (1.42, 2.15) | 848 (519, 1176) | 2758 (1318, 4198) | 14.98 (9.05, 20.90) | 25.37 (12.80, 37.94) | 1.69 (1.13, 2.26) |
| Bahamas | 0 (0, 0) | 1 (1, 2) | 0.25 (0.18, 0.32) | 0.32 (0.22, 0.42) | 0.77 (0.52, 1.02) | 84 (47, 121) | 287 (150, 423) | 48.03 (25.75, 70.31) | 69.85 (36.92, 102.78) | 1.30 (1.04, 1.55) |
| Bahrain | 1 (0, 1) | 6 (4, 8) | 0.47 (0.26, 0.68) | 1.13 (0.70, 1.56) | 2.79 (2.36, 3.22) | 110 (59, 161) | 1829 (907, 2752) | 60.79 (28.99, 92.59) | 206.32 (109.73, 302.92) | 3.98 (3.64, 4.32) |
| Bangladesh | 64 (21, 107) | 253 (156, 350) | 0.11 (0.07, 0.15) | 0.22 (0.13, 0.31) | 2.06 (1.81, 2.32) | 10364 (4830, 15899) | 47816 (23836, 71797) | 15.68 (8.67, 22.68) | 36.05 (17.52, 54.58) | 2.69 (2.54, 2.84) |
| Barbados | 1 (1, 2) | 3 (2, 4) | 0.41 (0.28, 0.53) | 0.52 (0.33, 0.70) | 0.54 (0.27, 0.81) | 198 (106, 291) | 508 (260, 755) | 70.99 (38.47, 103.51) | 106.03 (55.72, 156.34) | 1.09 (0.81, 1.37) |
| Belarus | 18 (15, 21) | 42 (32, 53) | 0.14 (0.12, 0.17) | 0.27 (0.21, 0.33) | 1.65 (1.48, 1.82) | 3285 (1996, 4574) | 9880 (5270, 14491) | 27.36 (16.64, 38.07) | 69.00 (37.96, 100.03) | 2.58 (2.41, 2.76) |
| Belgium | 56 (45, 67) | 95 (76, 114) | 0.37 (0.30, 0.43) | 0.37 (0.31, 0.43) | -0.11 (-0.29, 0.08) | 10979 (6638, 15319) | 23383 (14211, 32555) | 77.51 (47.13, 107.89) | 111.29 (69.34, 153.24) | 1.02 (0.82, 1.22) |
| Belize | 0 (0, 0) | 1 (1, 1) | 0.18 (0.13, 0.24) | 0.29 (0.20, 0.39) | 1.52 (1.16, 1.89) | 34 (20, 48) | 191 (107, 276) | 30.66 (16.79, 44.54) | 57.49 (31.56, 83.42) | 2.04 (1.73, 2.36) |
| Benin | 3 (2, 4) | 8 (5, 11) | 0.10 (0.07, 0.13) | 0.15 (0.10, 0.20) | 1.33 (1.27, 1.40) | 367 (216, 518) | 1410 (729, 2092) | 12.96 (7.46, 18.45) | 21.20 (10.65, 31.74) | 1.56 (1.49, 1.63) |
| Bermuda | 0 (0, 0) | 1 (0, 1) | 0.37 (0.27, 0.47) | 0.44 (0.33, 0.55) | 0.52 (0.40, 0.65) | 43 (24, 62) | 172 (96, 247) | 69.65 (39.09, 100.20) | 137.72 (78.32, 197.12) | 2.18 (1.96, 2.40) |
| Bhutan | 0 (0, 0) | 1 (1, 2) | 0.10 (0.06, 0.15) | 0.22 (0.14, 0.30) | 2.70 (2.62, 2.79) | 44 (21, 68) | 216 (113, 319) | 14.35 (6.51, 22.19) | 34.41 (18.11, 50.71) | 2.95 (2.85, 3.04) |
| Bolivia (Plurinational State of) | 4 (2, 5) | 16 (10, 23) | 0.11 (0.08, 0.14) | 0.20 (0.12, 0.28) | 2.04 (1.99, 2.09) | 683 (396, 970) | 3216 (1584, 4849) | 17.45 (9.83, 25.07) | 34.37 (16.87, 51.87) | 2.26 (2.20, 2.32) |
| Bosnia and Herzegovina | 7 (6, 9) | 29 (18, 39) | 0.19 (0.15, 0.24) | 0.45 (0.29, 0.60) | 2.94 (2.50, 3.38) | 1277 (775, 1780) | 4915 (2558, 7272) | 30.10 (18.41, 41.79) | 81.97 (44.50, 119.45) | 3.18 (2.45, 3.91) |
| Botswana | 1 (0, 1) | 3 (2, 4) | 0.16 (0.09, 0.24) | 0.24 (0.14, 0.34) | 1.47 (1.20, 1.73) | 148 (65, 232) | 544 (263, 826) | 22.86 (9.46, 36.25) | 34.52 (16.76, 52.28) | 1.60 (1.31, 1.89) |
| Brazil | 139 (105, 174) | 541 (409, 673) | 0.16 (0.12, 0.21) | 0.22 (0.17, 0.28) | 0.97 (0.87, 1.06) | 29846 (17577, 42115) | 112514 (67545, 157483) | 28.83 (16.87, 40.80) | 45.21 (27.23, 63.20) | 1.27 (1.15, 1.39) |
| Brunei Darussalam | 0 (0, 0) | 1 (1, 1) | 0.36 (0.21, 0.51) | 0.37 (0.26, 0.49) | 0.43 (0.21, 0.66) | 72 (37, 108) | 286 (152, 420) | 58.03 (24.74, 91.32) | 76.11 (41.62, 110.61) | 1.09 (0.89, 1.28) |
| Bulgaria | 30 (23, 37) | 52 (39, 65) | 0.27 (0.22, 0.33) | 0.37 (0.28, 0.46) | 0.54 (0.34, 0.73) | 4955 (2806, 7104) | 9007 (5091, 12924) | 44.44 (25.99, 62.88) | 73.76 (42.36, 105.15) | 1.25 (1.01, 1.49) |
| Burkina Faso | 6 (3, 9) | 16 (10, 21) | 0.12 (0.08, 0.17) | 0.16 (0.11, 0.21) | 0.79 (0.70, 0.89) | 934 (500, 1367) | 2757 (1472, 4042) | 16.21 (8.75, 23.68) | 22.08 (11.56, 32.60) | 1.01 (0.92, 1.09) |
| Burundi | 4 (2, 6) | 6 (4, 8) | 0.13 (0.09, 0.18) | 0.14 (0.09, 0.19) | -0.72 (-1.08, -0.35) | 559 (282, 837) | 1045 (545, 1546) | 17.40 (9.28, 25.52) | 16.93 (8.28, 25.59) | -1.56 (-2.25, -0.86) |
| Cabo Verde | 0 (0, 0) | 1 (1, 2) | 0.10 (0.08, 0.13) | 0.31 (0.21, 0.42) | 3.55 (3.38, 3.73) | 40 (24, 56) | 243 (129, 356) | 16.04 (9.67, 22.40) | 51.48 (27.73, 75.23) | 3.91 (3.79, 4.03) |
| Cambodia | 4 (3, 5) | 17 (10, 23) | 0.08 (0.06, 0.11) | 0.16 (0.10, 0.21) | 2.22 (2.00, 2.44) | 724 (405, 1043) | 3298 (1570, 5026) | 11.57 (6.11, 17.03) | 24.81 (12.34, 37.27) | 2.59 (2.36, 2.83) |
| Cameroon | 6 (4, 8) | 27 (16, 38) | 0.15 (0.10, 0.19) | 0.23 (0.14, 0.33) | 1.42 (1.34, 1.50) | 1052 (636, 1467) | 5300 (2634, 7966) | 19.29 (10.88, 27.71) | 33.93 (16.44, 51.41) | 1.72 (1.60, 1.84) |
| Canada | 132 (108, 156) | 307 (255, 359) | 0.41 (0.34, 0.48) | 0.41 (0.35, 0.47) | -0.31 (-0.64, 0.03) | 39416 (24060, 54772) | 105455 (65626, 145283) | 124.28 (76.00, 172.55) | 171.49 (107.09, 235.90) | 0.80 (0.45, 1.15) |
| Central African Republic | 1 (1, 2) | 3 (2, 4) | 0.11 (0.08, 0.15) | 0.13 (0.08, 0.18) | 0.49 (0.43, 0.55) | 227 (129, 326) | 561 (308, 814) | 14.99 (8.20, 21.78) | 18.15 (9.71, 26.58) | 0.60 (0.48, 0.72) |
| Chad | 3 (2, 4) | 8 (5, 11) | 0.09 (0.06, 0.11) | 0.13 (0.08, 0.17) | 1.33 (1.26, 1.39) | 448 (236, 660) | 1456 (819, 2092) | 11.23 (6.26, 16.20) | 17.10 (8.72, 25.49) | 1.43 (1.32, 1.54) |
| Chile | 16 (13, 19) | 66 (54, 78) | 0.16 (0.13, 0.20) | 0.25 (0.21, 0.30) | 1.62 (1.25, 1.99) | 3892 (2338, 5446) | 18045 (11118, 24971) | 35.92 (21.64, 50.21) | 73.89 (45.69, 102.09) | 2.60 (2.27, 2.94) |
| China | 632 (496, 767) | 5053 (3682, 6424) | 0.09 (0.07, 0.11) | 0.26 (0.19, 0.33) | 4.14 (3.87, 4.41) | 121640 (74136, 169144) | 1366971 (720447, 2013494) | 13.41 (8.10, 18.71) | 70.00 (37.53, 102.46) | 6.28 (5.96, 6.59) |
| Colombia | 25 (20, 29) | 107 (79, 134) | 0.14 (0.11, 0.16) | 0.19 (0.14, 0.24) | 0.51 (0.24, 0.77) | 5945 (3620, 8271) | 29620 (16418, 42821) | 26.58 (16.20, 36.95) | 54.21 (30.02, 78.40) | 1.75 (1.48, 2.02) |
| Comoros | 0 (0, 1) | 1 (1, 1) | 0.17 (0.10, 0.23) | 0.21 (0.13, 0.29) | 0.64 (0.45, 0.82) | 58 (27, 89) | 154 (77, 231) | 22.38 (11.00, 33.76) | 29.08 (14.52, 43.65) | 0.73 (0.50, 0.97) |
| Congo | 2 (1, 2) | 6 (4, 8) | 0.17 (0.11, 0.23) | 0.26 (0.17, 0.35) | 1.15 (0.86, 1.44) | 291 (158, 423) | 1181 (561, 1801) | 23.44 (12.48, 34.40) | 37.79 (19.19, 56.39) | 1.19 (0.70, 1.68) |
| Cook Islands | 0 (0, 0) | 0 (0, 0) | 0.60 (0.29, 0.91) | 0.88 (0.50, 1.25) | 0.99 (0.77, 1.21) | 13 (5, 21) | 49 (21, 78) | 98.02 (35.58, 160.45) | 198.67 (86.05, 311.29) | 2.12 (1.93, 2.31) |
| Costa Rica | 3 (2, 4) | 15 (12, 19) | 0.17 (0.13, 0.20) | 0.28 (0.21, 0.34) | 1.21 (0.90, 1.52) | 718 (430, 1007) | 4403 (2611, 6194) | 36.03 (21.35, 50.70) | 80.78 (47.92, 113.64) | 2.28 (1.97, 2.58) |
| Côte d'Ivoire | 6 (4, 7) | 21 (13, 28) | 0.13 (0.09, 0.18) | 0.20 (0.13, 0.28) | 1.32 (1.25, 1.39) | 949 (528, 1369) | 3954 (1959, 5949) | 16.86 (8.86, 24.86) | 28.75 (14.40, 43.10) | 1.64 (1.57, 1.71) |
| Croatia | 20 (17, 24) | 45 (35, 56) | 0.37 (0.30, 0.44) | 0.48 (0.38, 0.58) | 0.68 (0.46, 0.89) | 3713 (2257, 5168) | 8928 (5338, 12518) | 63.80 (39.64, 87.97) | 107.06 (65.28, 148.84) | 1.44 (1.16, 1.72) |
| Cuba | 22 (16, 27) | 57 (45, 70) | 0.22 (0.17, 0.27) | 0.28 (0.22, 0.34) | 0.71 (0.47, 0.96) | 4156 (2432, 5879) | 12356 (6980, 17732) | 40.37 (23.40, 57.33) | 69.08 (39.21, 98.95) | 1.47 (1.24, 1.71) |
| Cyprus | 4 (3, 6) | 11 (8, 15) | 0.79 (0.46, 1.11) | 0.63 (0.47, 0.78) | -0.72 (-0.85, -0.59) | 653 (316, 989) | 2842 (1588, 4097) | 103.60 (49.44, 157.75) | 154.87 (86.77, 222.97) | 1.60 (1.30, 1.89) |
| Czechia | 49 (38, 59) | 108 (80, 136) | 0.36 (0.29, 0.43) | 0.48 (0.36, 0.60) | 1.18 (1.09, 1.28) | 7863 (4594, 11131) | 21589 (12620, 30558) | 59.04 (35.11, 82.97) | 109.12 (64.30, 153.94) | 2.12 (1.88, 2.37) |
| Democratic People's Republic of Korea | 14 (9, 18) | 48 (33, 64) | 0.09 (0.06, 0.12) | 0.15 (0.11, 0.20) | -1.34 (-3.65, 1.02) | 2830 (1470, 4190) | 10883 (5121, 16645) | 16.16 (8.53, 23.79) | 34.02 (16.13, 51.90) | 0.00 (-1.87, 1.91) |
| Democratic Republic of the Congo | 20 (11, 28) | 54 (36, 72) | 0.12 (0.08, 0.17) | 0.16 (0.10, 0.23) | 0.78 (0.46, 1.10) | 3096 (1648, 4545) | 10269 (5374, 15164) | 15.46 (8.20, 22.72) | 22.79 (11.56, 34.02) | 1.04 (0.65, 1.44) |
| Denmark | 31 (25, 37) | 53 (43, 64) | 0.37 (0.30, 0.43) | 0.42 (0.34, 0.49) | 0.37 (0.06, 0.68) | 7027 (4064, 9990) | 15027 (8799, 21254) | 94.15 (54.77, 133.54) | 136.61 (81.40, 191.82) | 1.36 (1.04, 1.69) |
| Djibouti | 0 (0, 0) | 1 (1, 1) | 0.13 (0.08, 0.18) | 0.21 (0.12, 0.29) | 1.39 (1.27, 1.50) | 37 (19, 56) | 210 (95, 324) | 17.81 (9.06, 26.57) | 28.71 (13.85, 43.56) | 1.54 (1.39, 1.69) |
| Dominica | 0 (0, 0) | 0 (0, 0) | 0.27 (0.17, 0.36) | 0.37 (0.23, 0.50) | 0.80 (0.66, 0.93) | 24 (12, 36) | 51 (26, 76) | 41.52 (21.19, 61.84) | 63.76 (33.02, 94.50) | 1.21 (1.09, 1.34) |
| Dominican Republic | 4 (3, 6) | 19 (12, 26) | 0.12 (0.08, 0.15) | 0.19 (0.13, 0.26) | 2.00 (1.73, 2.29) | 875 (511, 1240) | 4172 (2093, 6250) | 19.15 (10.80, 27.50) | 39.89 (20.08, 59.69) | 2.71 (2.45, 2.96) |
| Ecuador | 6 (4, 7) | 35 (23, 46) | 0.11 (0.08, 0.13) | 0.22 (0.15, 0.29) | 2.46 (2.01, 2.91) | 1202 (721, 1682) | 7897 (4097, 11697) | 18.96 (11.07, 26.86) | 47.71 (24.80, 70.61) | 2.95 (2.47, 3.44) |
| Egypt | 27 (22, 33) | 196 (123, 269) | 0.11 (0.09, 0.14) | 0.39 (0.24, 0.54) | 4.37 (4.10, 4.63) | 4996 (3099, 6894) | 40761 (21138, 60384) | 15.57 (9.38, 21.75) | 61.96 (33.18, 90.73) | 4.70 (4.50, 4.90) |
| El Salvador | 4 (3, 4) | 20 (13, 27) | 0.10 (0.08, 0.13) | 0.32 (0.21, 0.44) | 3.73 (3.35, 4.11) | 779 (461, 1097) | 5171 (2748, 7595) | 20.66 (12.34, 28.98) | 83.30 (43.87, 122.74) | 4.78 (4.38, 5.18) |
| Equatorial Guinea | 0 (0, 0) | 2 (1, 2) | 0.12 (0.07, 0.17) | 0.37 (0.21, 0.53) | 4.13 (3.91, 4.35) | 38 (19, 58) | 381 (160, 602) | 16.02 (7.64, 24.41) | 57.61 (25.88, 89.33) | 4.72 (4.46, 4.98) |
| Eritrea | 3 (1, 5) | 4 (3, 5) | 0.17 (0.11, 0.24) | 0.17 (0.11, 0.23) | 0.15 (-0.11, 0.41) | 728 (59, 1397) | 768 (373, 1163) | 27.30 (9.76, 44.84) | 21.62 (11.01, 32.23) | -0.01 (-0.53, 0.52) |
| Estonia | 4 (3, 5) | 10 (8, 12) | 0.21 (0.17, 0.24) | 0.37 (0.30, 0.43) | 1.34 (1.00, 1.68) | 786 (467, 1104) | 2191 (1314, 3069) | 41.94 (25.01, 58.86) | 96.75 (58.90, 134.59) | 2.29 (1.93, 2.65) |
| Eswatini | 1 (0, 1) | 2 (1, 3) | 0.19 (0.11, 0.27) | 0.33 (0.17, 0.50) | 2.17 (1.81, 2.53) | 97 (49, 145) | 341 (145, 536) | 26.91 (12.41, 41.42) | 51.88 (21.43, 82.34) | 2.46 (2.06, 2.85) |
| Ethiopia | 33 (19, 47) | 70 (52, 87) | 0.14 (0.09, 0.18) | 0.16 (0.11, 0.21) | 0.48 (0.26, 0.70) | 6268 (2742, 9794) | 12387 (8045, 16729) | 18.96 (10.41, 27.50) | 21.70 (13.15, 30.26) | 0.46 (0.13, 0.80) |
| Fiji | 1 (1, 2) | 5 (2, 8) | 0.44 (0.20, 0.68) | 0.75 (0.35, 1.15) | 1.37 (1.18, 1.56) | 273 (110, 436) | 907 (304, 1509) | 68.34 (24.42, 112.25) | 117.69 (44.44, 190.94) | 1.47 (1.32, 1.62) |
| Finland | 22 (18, 26) | 46 (37, 55) | 0.31 (0.26, 0.36) | 0.34 (0.28, 0.39) | 0.38 (0.14, 0.62) | 4303 (2669, 5936) | 10997 (6622, 15371) | 64.83 (40.13, 89.53) | 102.38 (63.53, 141.24) | 1.66 (1.35, 1.98) |
| France | 365 (292, 438) | 768 (604, 931) | 0.42 (0.35, 0.50) | 0.46 (0.38, 0.54) | 0.39 (0.07, 0.70) | 63039 (37699, 88379) | 177576 (104252, 250900) | 80.47 (48.50, 112.45) | 134.69 (83.06, 186.32) | 1.87 (1.54, 2.19) |
| Gabon | 1 (1, 2) | 3 (2, 5) | 0.20 (0.12, 0.28) | 0.38 (0.24, 0.53) | 1.94 (1.85, 2.03) | 164 (83, 246) | 651 (312, 990) | 27.23 (13.26, 41.21) | 57.24 (28.64, 85.85) | 2.31 (2.19, 2.42) |
| Gambia | 0 (0, 1) | 2 (1, 3) | 0.12 (0.08, 0.17) | 0.22 (0.14, 0.30) | 1.78 (1.67, 1.89) | 80 (43, 118) | 376 (184, 568) | 16.39 (8.46, 24.31) | 31.59 (14.93, 48.25) | 2.00 (1.84, 2.16) |
| Georgia | 8 (7, 10) | 13 (9, 16) | 0.14 (0.11, 0.17) | 0.21 (0.16, 0.27) | 1.85 (1.47, 2.23) | 1526 (913, 2138) | 2023 (1149, 2897) | 25.50 (15.42, 35.57) | 37.92 (22.45, 53.40) | 1.40 (1.08, 1.72) |
| Germany | 452 (349, 555) | 989 (787, 1192) | 0.35 (0.28, 0.42) | 0.46 (0.38, 0.54) | 0.84 (0.66, 1.01) | 86494 (50670, 122319) | 244707 (145382, 344032) | 73.49 (43.65, 103.33) | 139.73 (85.26, 194.21) | 1.84 (1.50, 2.19) |
| Ghana | 9 (5, 13) | 38 (26, 50) | 0.14 (0.09, 0.18) | 0.26 (0.17, 0.34) | 2.21 (2.07, 2.35) | 1603 (862, 2344) | 7380 (3796, 10964) | 19.30 (10.18, 28.43) | 38.60 (19.62, 57.57) | 2.34 (2.22, 2.46) |
| Greece | 40 (33, 47) | 82 (67, 98) | 0.27 (0.22, 0.32) | 0.31 (0.26, 0.36) | 0.18 (0.02, 0.35) | 8931 (5340, 12521) | 19992 (12290, 27694) | 64.94 (38.63, 91.25) | 95.17 (59.57, 130.77) | 1.01 (0.82, 1.20) |
| Greenland | 0 (0, 0) | 0 (0, 0) | 0.24 (0.19, 0.29) | 0.25 (0.18, 0.31) | 0.33 (0.25, 0.40) | 18 (10, 25) | 40 (22, 57) | 42.66 (25.15, 60.17) | 60.24 (34.76, 85.73) | 1.41 (1.33, 1.49) |
| Grenada | 0 (0, 0) | 1 (0, 1) | 0.31 (0.20, 0.41) | 0.50 (0.35, 0.65) | 1.30 (0.86, 1.74) | 37 (19, 54) | 98 (54, 143) | 50.19 (26.27, 74.11) | 90.82 (50.05, 131.59) | 1.49 (1.15, 1.83) |
| Guam | 0 (0, 0) | 0 (0, 0) | 0.26 (0.17, 0.35) | 0.16 (0.11, 0.21) | -0.89 (-1.15, -0.63) | 38 (20, 57) | 86 (46, 127) | 46.17 (23.94, 68.39) | 43.70 (23.68, 63.73) | 0.55 (0.24, 0.86) |
| Guatemala | 6 (5, 8) | 30 (19, 40) | 0.19 (0.14, 0.24) | 0.28 (0.18, 0.38) | 0.88 (0.66, 1.10) | 1251 (715, 1786) | 6782 (3533, 10031) | 28.69 (16.90, 40.49) | 56.44 (29.33, 83.54) | 1.88 (1.65, 2.11) |
| Guinea | 3 (2, 4) | 8 (5, 12) | 0.08 (0.06, 0.11) | 0.15 (0.09, 0.20) | 1.84 (1.70, 1.98) | 453 (247, 660) | 1425 (713, 2137) | 10.90 (5.87, 15.92) | 20.06 (9.78, 30.34) | 1.98 (1.85, 2.12) |
| Guinea-Bissau | 1 (0, 1) | 1 (1, 2) | 0.12 (0.08, 0.16) | 0.18 (0.12, 0.24) | 1.30 (1.24, 1.37) | 95 (50, 141) | 251 (131, 370) | 16.64 (8.66, 24.62) | 25.61 (13.34, 37.88) | 1.40 (1.29, 1.51) |
| Guyana | 1 (0, 1) | 2 (1, 2) | 0.19 (0.13, 0.26) | 0.29 (0.16, 0.42) | 1.06 (0.73, 1.38) | 139 (75, 203) | 338 (155, 521) | 30.32 (15.54, 45.10) | 50.65 (23.76, 77.53) | 1.40 (1.06, 1.73) |
| Haiti | 4 (2, 6) | 10 (6, 15) | 0.12 (0.07, 0.17) | 0.17 (0.09, 0.24) | 1.09 (0.71, 1.47) | 685 (352, 1017) | 1955 (882, 3028) | 17.51 (8.61, 26.41) | 23.85 (10.27, 37.43) | 1.14 (0.71, 1.57) |
| Honduras | 2 (1, 2) | 9 (6, 13) | 0.07 (0.05, 0.09) | 0.16 (0.11, 0.21) | 2.83 (2.49, 3.17) | 383 (231, 536) | 2061 (1063, 3059) | 13.63 (8.28, 18.98) | 30.00 (16.18, 43.81) | 2.65 (2.39, 2.91) |
| Hungary | 53 (42, 63) | 85 (68, 101) | 0.38 (0.30, 0.45) | 0.42 (0.34, 0.51) | -0.04 (-0.30, 0.23) | 8113 (4848, 11379) | 17828 (10505, 25151) | 59.33 (35.79, 82.88) | 100.57 (60.21, 140.92) | 1.21 (0.86, 1.57) |
| Iceland | 1 (1, 1) | 2 (2, 2) | 0.30 (0.25, 0.35) | 0.31 (0.25, 0.37) | 0.03 (-0.16, 0.22) | 220 (135, 305) | 573 (350, 795) | 79.17 (48.66, 109.67) | 107.07 (66.48, 147.67) | 0.77 (0.56, 0.99) |
| India | 441 (299, 582) | 2497 (1879, 3115) | 0.10 (0.07, 0.14) | 0.24 (0.18, 0.30) | 2.81 (2.54, 3.09) | 83394 (47706, 119081) | 488024 (286497, 689550) | 15.26 (8.50, 22.02) | 39.98 (23.72, 56.23) | 3.26 (3.03, 3.49) |
| Indonesia | 70 (51, 88) | 322 (216, 428) | 0.08 (0.06, 0.10) | 0.17 (0.11, 0.22) | 2.43 (2.37, 2.50) | 13493 (7799, 19187) | 65348 (32886, 97810) | 11.43 (6.42, 16.43) | 26.56 (13.81, 39.31) | 2.67 (2.59, 2.75) |
| Iran (Islamic Republic of) | 33 (27, 40) | 198 (150, 245) | 0.12 (0.10, 0.15) | 0.28 (0.21, 0.35) | 3.17 (3.03, 3.31) | 6420 (3985, 8855) | 44625 (27617, 61634) | 18.35 (11.18, 25.53) | 55.08 (34.35, 75.80) | 4.06 (3.89, 4.23) |
| Iraq | 15 (10, 20) | 84 (51, 116) | 0.17 (0.11, 0.24) | 0.40 (0.24, 0.55) | 2.93 (2.65, 3.20) | 2766 (1593, 3939) | 19134 (9032, 29235) | 26.35 (13.73, 38.97) | 70.52 (34.15, 106.90) | 3.86 (3.46, 4.27) |
| Ireland | 15 (12, 18) | 28 (23, 33) | 0.37 (0.31, 0.44) | 0.34 (0.28, 0.40) | -0.03 (-0.24, 0.17) | 2953 (1824, 4082) | 7893 (4837, 10949) | 75.40 (46.39, 104.41) | 105.36 (65.26, 145.46) | 1.49 (1.18, 1.80) |
| Israel | 17 (13, 20) | 60 (46, 73) | 0.35 (0.28, 0.43) | 0.45 (0.36, 0.55) | 0.58 (0.01, 1.15) | 3091 (1841, 4341) | 14773 (8945, 20602) | 65.91 (39.63, 92.19) | 122.68 (75.28, 170.07) | 1.81 (1.18, 2.45) |
| Italy | 367 (272, 462) | 640 (492, 789) | 0.41 (0.31, 0.51) | 0.38 (0.30, 0.45) | -0.35 (-0.58, -0.12) | 72113 (41492, 102734) | 152161 (89826, 214495) | 85.83 (50.41, 121.24) | 111.89 (68.98, 154.80) | 0.80 (0.42, 1.17) |
| Jamaica | 6 (4, 8) | 14 (9, 20) | 0.32 (0.21, 0.42) | 0.44 (0.26, 0.63) | 0.89 (0.57, 1.22) | 956 (500, 1412) | 2668 (1354, 3983) | 52.60 (27.08, 78.12) | 84.25 (42.60, 125.89) | 1.21 (0.81, 1.61) |
| Japan | 354 (295, 413) | 915 (725, 1104) | 0.22 (0.18, 0.26) | 0.23 (0.19, 0.27) | 0.08 (-0.01, 0.18) | 97562 (59968, 135155) | 258406 (153570, 363241) | 60.36 (37.18, 83.54) | 88.64 (55.17, 122.11) | 1.19 (1.09, 1.29) |
| Jordan | 3 (2, 4) | 23 (15, 30) | 0.23 (0.13, 0.34) | 0.37 (0.24, 0.50) | 1.48 (1.12, 1.84) | 554 (301, 807) | 6008 (3057, 8960) | 34.72 (16.86, 52.57) | 74.54 (38.52, 110.55) | 2.71 (2.33, 3.08) |
| Kazakhstan | 15 (13, 18) | 32 (26, 38) | 0.12 (0.10, 0.14) | 0.19 (0.16, 0.23) | 1.58 (1.31, 1.84) | 2954 (1788, 4120) | 7090 (4154, 10026) | 21.30 (12.94, 29.67) | 38.13 (22.57, 53.68) | 2.03 (1.68, 2.38) |
| Kenya | 9 (6, 13) | 34 (24, 45) | 0.11 (0.07, 0.14) | 0.18 (0.12, 0.23) | 1.37 (1.24, 1.50) | 1486 (882, 2091) | 6229 (3486, 8972) | 13.45 (7.59, 19.31) | 23.60 (13.31, 33.89) | 1.65 (1.52, 1.77) |
| Kiribati | 0 (0, 0) | 0 (0, 0) | 0.19 (0.10, 0.29) | 0.31 (0.16, 0.46) | 1.44 (1.28, 1.59) | 12 (5, 18) | 35 (13, 57) | 27.80 (11.79, 43.80) | 46.13 (18.74, 73.52) | 1.51 (1.34, 1.68) |
| Kuwait | 2 (1, 3) | 10 (7, 13) | 0.27 (0.20, 0.35) | 0.40 (0.27, 0.53) | 1.37 (0.90, 1.85) | 714 (301, 1127) | 3585 (1868, 5301) | 65.95 (35.60, 96.29) | 99.38 (52.68, 146.08) | 1.97 (1.43, 2.51) |
| Kyrgyzstan | 3 (3, 4) | 7 (6, 9) | 0.10 (0.09, 0.12) | 0.16 (0.13, 0.20) | 1.77 (1.35, 2.18) | 614 (381, 846) | 1706 (931, 2480) | 18.43 (11.36, 25.49) | 31.18 (17.21, 45.14) | 1.86 (1.45, 2.27) |
| Lao People's Democratic Republic | 2 (1, 2) | 6 (4, 8) | 0.08 (0.05, 0.11) | 0.14 (0.09, 0.19) | 1.89 (1.79, 1.99) | 339 (192, 486) | 1258 (562, 1953) | 12.46 (6.76, 18.16) | 23.64 (10.98, 36.31) | 2.18 (2.07, 2.30) |
| Latvia | 8 (6, 9) | 14 (11, 17) | 0.22 (0.19, 0.25) | 0.35 (0.28, 0.41) | 0.81 (0.39, 1.24) | 1403 (850, 1956) | 2680 (1542, 3818) | 43.04 (26.10, 59.98) | 81.89 (48.28, 115.50) | 1.19 (0.66, 1.72) |
| Lebanon | 5 (4, 7) | 25 (17, 34) | 0.27 (0.17, 0.36) | 0.40 (0.27, 0.53) | 1.86 (1.67, 2.05) | 955 (532, 1379) | 5241 (3086, 7397) | 40.86 (22.33, 59.40) | 85.82 (50.06, 121.58) | 3.26 (3.02, 3.50) |
| Lesotho | 1 (1, 1) | 2 (2, 3) | 0.12 (0.07, 0.17) | 0.24 (0.15, 0.34) | 3.00 (2.68, 3.33) | 151 (75, 227) | 442 (206, 678) | 15.82 (7.44, 24.20) | 36.24 (17.03, 55.46) | 3.24 (2.88, 3.60) |
| Liberia | 2 (1, 3) | 4 (2, 6) | 0.13 (0.09, 0.18) | 0.20 (0.12, 0.29) | 1.76 (1.55, 1.97) | 416 (86, 747) | 794 (379, 1209) | 21.47 (8.33, 34.62) | 28.78 (13.44, 44.13) | 1.71 (1.23, 2.19) |
| Libya | 3 (2, 3) | 15 (10, 21) | 0.13 (0.09, 0.16) | 0.31 (0.20, 0.42) | 4.02 (3.62, 4.42) | 529 (294, 764) | 3984 (2063, 5904) | 21.38 (11.83, 30.93) | 63.97 (33.44, 94.49) | 4.89 (4.38, 5.40) |
| Lithuania | 9 (7, 10) | 19 (15, 22) | 0.20 (0.17, 0.23) | 0.33 (0.27, 0.39) | 1.02 (0.72, 1.33) | 1734 (1047, 2421) | 3670 (2083, 5257) | 40.91 (24.73, 57.08) | 77.58 (44.72, 110.44) | 1.26 (0.91, 1.62) |
| Luxembourg | 2 (1, 2) | 4 (3, 5) | 0.34 (0.28, 0.40) | 0.35 (0.28, 0.42) | 0.21 (-0.04, 0.47) | 348 (213, 483) | 1039 (613, 1465) | 68.87 (42.26, 95.49) | 99.22 (59.53, 138.91) | 1.30 (0.91, 1.70) |
| Madagascar | 7 (3, 11) | 14 (9, 18) | 0.12 (0.08, 0.16) | 0.13 (0.09, 0.18) | 0.26 (0.16, 0.36) | 1164 (588, 1740) | 2797 (1528, 4067) | 16.06 (9.45, 22.67) | 18.66 (10.15, 27.16) | 0.43 (0.32, 0.54) |
| Malawi | 6 (3, 9) | 13 (9, 17) | 0.12 (0.08, 0.17) | 0.18 (0.12, 0.25) | 1.07 (0.94, 1.21) | 877 (457, 1296) | 2491 (1318, 3664) | 15.72 (8.55, 22.89) | 26.00 (13.57, 38.42) | 1.47 (1.32, 1.61) |
| Malaysia | 12 (9, 15) | 57 (45, 69) | 0.13 (0.09, 0.17) | 0.21 (0.17, 0.26) | 1.59 (1.39, 1.78) | 2497 (1495, 3499) | 14521 (8708, 20333) | 22.52 (13.03, 32.00) | 47.99 (29.10, 66.88) | 2.44 (2.28, 2.61) |
| Maldives | 0 (0, 0) | 1 (0, 1) | 0.13 (0.09, 0.18) | 0.20 (0.14, 0.27) | 1.25 (1.06, 1.44) | 24 (14, 35) | 182 (97, 268) | 21.08 (10.75, 31.40) | 43.32 (23.41, 63.22) | 2.37 (2.16, 2.58) |
| Mali | 5 (3, 8) | 17 (10, 24) | 0.11 (0.08, 0.14) | 0.17 (0.12, 0.23) | 1.52 (1.41, 1.64) | 781 (436, 1126) | 2760 (1525, 3996) | 14.12 (8.04, 20.20) | 22.72 (12.00, 33.44) | 1.53 (1.29, 1.77) |
| Malta | 2 (1, 2) | 4 (3, 5) | 0.38 (0.28, 0.47) | 0.40 (0.31, 0.49) | 0.23 (0.03, 0.44) | 281 (165, 396) | 980 (586, 1373) | 67.77 (40.18, 95.35) | 110.50 (67.29, 153.70) | 1.60 (1.37, 1.83) |
| Marshall Islands | 0 (0, 0) | 0 (0, 0) | 0.20 (0.10, 0.31) | 0.37 (0.17, 0.57) | 1.99 (1.86, 2.11) | 6 (3, 9) | 23 (8, 38) | 30.28 (11.74, 48.82) | 58.67 (22.18, 95.16) | 2.19 (2.06, 2.31) |
| Mauritania | 1 (1, 2) | 5 (3, 6) | 0.13 (0.09, 0.17) | 0.24 (0.15, 0.33) | 1.77 (1.57, 1.97) | 208 (121, 296) | 854 (436, 1273) | 17.36 (9.72, 25.00) | 35.74 (17.64, 53.85) | 2.09 (1.86, 2.32) |
| Mauritius | 2 (1, 3) | 14 (9, 19) | 0.27 (0.18, 0.37) | 0.78 (0.51, 1.04) | 4.33 (3.67, 5.00) | 456 (240, 671) | 3250 (1548, 4952) | 55.22 (28.97, 81.47) | 179.80 (90.75, 268.86) | 4.60 (4.08, 5.12) |
| Mexico | 105 (74, 137) | 500 (335, 666) | 0.26 (0.18, 0.34) | 0.41 (0.27, 0.55) | 1.32 (1.18, 1.46) | 22415 (12681, 32148) | 128185 (64715, 191654) | 46.07 (24.71, 67.43) | 98.56 (50.13, 147.00) | 2.19 (2.01, 2.36) |
| Micronesia (Federated States of) | 0 (0, 0) | 0 (0, 0) | 0.18 (0.10, 0.26) | 0.34 (0.17, 0.50) | 2.20 (1.98, 2.41) | 15 (6, 23) | 44 (16, 72) | 26.49 (10.74, 42.25) | 54.97 (21.91, 88.04) | 2.49 (2.28, 2.69) |
| Monaco | 0 (0, 0) | 0 (0, 0) | 0.31 (0.23, 0.40) | 0.37 (0.28, 0.46) | 0.61 (0.46, 0.76) | 51 (28, 75) | 103 (59, 147) | 85.87 (47.54, 124.20) | 130.95 (73.99, 187.91) | 1.45 (1.27, 1.64) |
| Mongolia | 1 (1, 1) | 3 (2, 4) | 0.06 (0.05, 0.08) | 0.14 (0.11, 0.18) | 3.14 (2.83, 3.45) | 131 (84, 177) | 645 (398, 892) | 9.51 (6.12, 12.90) | 24.04 (14.67, 33.41) | 3.61 (3.24, 3.98) |
| Montenegro | 1 (1, 2) | 4 (3, 5) | 0.24 (0.19, 0.30) | 0.40 (0.30, 0.50) | 2.11 (1.89, 2.33) | 314 (187, 442) | 797 (460, 1134) | 50.05 (29.81, 70.29) | 88.24 (51.98, 124.50) | 2.55 (2.32, 2.79) |
| Morocco | 11 (9, 14) | 62 (42, 81) | 0.07 (0.06, 0.09) | 0.20 (0.14, 0.26) | 3.43 (3.29, 3.58) | 1982 (1211, 2753) | 11508 (6118, 16897) | 11.25 (6.85, 15.65) | 32.89 (17.98, 47.79) | 3.69 (3.55, 3.84) |
| Mozambique | 7 (4, 10) | 17 (11, 23) | 0.10 (0.07, 0.13) | 0.15 (0.10, 0.21) | 1.68 (1.59, 1.77) | 1044 (551, 1537) | 3220 (1566, 4874) | 12.39 (6.83, 17.94) | 21.46 (10.53, 32.38) | 2.14 (2.02, 2.25) |
| Myanmar | 23 (15, 32) | 91 (58, 124) | 0.11 (0.07, 0.15) | 0.21 (0.13, 0.29) | 2.06 (1.88, 2.25) | 4596 (2509, 6682) | 17724 (8208, 27241) | 16.64 (8.84, 24.44) | 34.92 (16.61, 53.22) | 2.32 (2.15, 2.49) |
| Namibia | 1 (1, 1) | 4 (2, 6) | 0.16 (0.10, 0.23) | 0.31 (0.18, 0.45) | 1.98 (1.81, 2.15) | 171 (76, 265) | 730 (322, 1138) | 22.35 (9.63, 35.07) | 48.17 (20.90, 75.43) | 2.33 (2.13, 2.54) |
| Nauru | 0 (0, 0) | 0 (0, 0) | 0.30 (0.15, 0.45) | 0.47 (0.26, 0.68) | 1.34 (1.11, 1.58) | 3 (1, 4) | 5 (2, 9) | 47.15 (20.54, 73.77) | 80.59 (33.41, 127.76) | 1.57 (1.29, 1.85) |
| Nepal | 9 (4, 14) | 38 (25, 51) | 0.09 (0.06, 0.12) | 0.19 (0.13, 0.26) | 2.65 (2.37, 2.93) | 1528 (753, 2303) | 6802 (3484, 10119) | 12.55 (6.67, 18.42) | 29.11 (15.21, 43.02) | 2.88 (2.59, 3.16) |
| Netherlands | 91 (72, 111) | 184 (146, 222) | 0.45 (0.36, 0.54) | 0.48 (0.39, 0.57) | 0.30 (0.19, 0.40) | 20202 (12126, 28279) | 45946 (27448, 64444) | 104.49 (63.18, 145.79) | 138.45 (84.81, 192.09) | 1.10 (0.89, 1.31) |
| New Zealand | 14 (11, 16) | 34 (28, 41) | 0.36 (0.29, 0.42) | 0.39 (0.32, 0.46) | 0.22 (0.05, 0.39) | 2673 (1633, 3714) | 8322 (5067, 11578) | 69.87 (42.70, 97.05) | 104.67 (65.02, 144.33) | 1.28 (1.11, 1.45) |
| Nicaragua | 2 (1, 2) | 10 (7, 13) | 0.11 (0.08, 0.14) | 0.21 (0.14, 0.28) | 2.41 (2.17, 2.65) | 415 (255, 575) | 2604 (1452, 3756) | 19.72 (11.55, 27.88) | 48.36 (27.12, 69.60) | 3.24 (3.02, 3.46) |
| Niger | 3 (1, 5) | 10 (6, 13) | 0.08 (0.05, 0.10) | 0.11 (0.07, 0.15) | 1.31 (1.21, 1.40) | 488 (233, 743) | 1660 (894, 2426) | 9.85 (5.43, 14.27) | 14.53 (7.40, 21.66) | 1.37 (1.23, 1.51) |
| Nigeria | 56 (39, 72) | 172 (114, 230) | 0.13 (0.09, 0.17) | 0.21 (0.14, 0.29) | 1.64 (1.47, 1.80) | 8308 (4919, 11698) | 29628 (16776, 42480) | 16.28 (8.98, 23.58) | 27.79 (15.56, 40.02) | 1.79 (1.61, 1.98) |
| Niue | 0 (0, 0) | 0 (0, 0) | 0.28 (0.15, 0.42) | 0.60 (0.32, 0.89) | 2.35 (2.15, 2.55) | 1 (0, 2) | 2 (1, 4) | 45.43 (16.85, 74.02) | 109.27 (45.53, 173.01) | 2.69 (2.49, 2.88) |
| North Macedonia | 3 (3, 4) | 12 (8, 16) | 0.20 (0.15, 0.25) | 0.42 (0.29, 0.55) | 2.61 (2.12, 3.09) | 542 (325, 759) | 2184 (1147, 3220) | 28.72 (17.25, 40.18) | 71.59 (38.50, 104.68) | 3.34 (2.85, 3.83) |
| Northern Mariana Islands | 0 (0, 0) | 0 (0, 0) | 0.36 (0.21, 0.52) | 0.44 (0.31, 0.58) | 0.32 (0.02, 0.62) | 16 (7, 25) | 47 (23, 72) | 66.03 (29.27, 102.79) | 88.53 (46.24, 130.82) | 0.53 (0.20, 0.86) |
| Norway | 24 (19, 29) | 43 (35, 52) | 0.33 (0.27, 0.40) | 0.39 (0.32, 0.46) | 0.50 (0.22, 0.78) | 4291 (2622, 5960) | 10493 (6303, 14684) | 67.91 (41.97, 93.86) | 110.38 (68.28, 152.47) | 1.60 (1.16, 2.04) |
| Oman | 2 (1, 3) | 10 (7, 13) | 0.27 (0.15, 0.39) | 0.60 (0.39, 0.81) | 2.86 (2.63, 3.10) | 378 (179, 578) | 3190 (1692, 4688) | 40.46 (18.21, 62.72) | 122.97 (66.36, 179.58) | 3.86 (3.61, 4.12) |
| Pakistan | 56 (38, 75) | 208 (141, 274) | 0.10 (0.06, 0.14) | 0.20 (0.13, 0.27) | 2.06 (1.97, 2.14) | 8792 (5172, 12412) | 39305 (23256, 55355) | 13.43 (7.37, 19.49) | 29.03 (16.89, 41.16) | 2.36 (2.29, 2.44) |
| Palau | 0 (0, 0) | 0 (0, 0) | 0.36 (0.20, 0.53) | 0.59 (0.37, 0.81) | 1.60 (1.46, 1.75) | 7 (3, 10) | 24 (11, 38) | 60.50 (24.81, 96.20) | 109.14 (52.26, 166.01) | 1.88 (1.71, 2.05) |
| Palestine | 2 (1, 3) | 8 (5, 11) | 0.23 (0.12, 0.34) | 0.40 (0.25, 0.54) | 1.80 (1.47, 2.12) | 296 (142, 451) | 1679 (912, 2446) | 28.94 (12.77, 45.11) | 62.27 (32.07, 92.47) | 2.45 (1.96, 2.95) |
| Panama | 2 (2, 3) | 13 (8, 17) | 0.15 (0.12, 0.19) | 0.28 (0.18, 0.38) | 1.73 (1.51, 1.95) | 500 (303, 696) | 3249 (1624, 4874) | 29.73 (17.79, 41.67) | 73.40 (36.56, 110.24) | 2.61 (2.41, 2.82) |
| Papua New Guinea | 3 (1, 4) | 9 (5, 13) | 0.18 (0.08, 0.27) | 0.21 (0.11, 0.31) | 0.48 (0.41, 0.56) | 540 (191, 890) | 1882 (806, 2958) | 25.29 (8.43, 42.14) | 31.70 (13.24, 50.16) | 0.53 (0.42, 0.63) |
| Paraguay | 2 (2, 3) | 20 (11, 29) | 0.11 (0.07, 0.15) | 0.36 (0.20, 0.52) | 4.06 (3.78, 4.34) | 517 (303, 730) | 4433 (1991, 6875) | 20.00 (11.28, 28.72) | 72.58 (32.69, 112.47) | 4.31 (4.06, 4.56) |
| Peru | 12 (9, 14) | 67 (44, 91) | 0.09 (0.07, 0.11) | 0.20 (0.13, 0.27) | 2.54 (2.27, 2.82) | 2576 (1531, 3621) | 18508 (9491, 27525) | 16.86 (10.03, 23.68) | 52.77 (27.05, 78.50) | 3.67 (3.32, 4.02) |
| Philippines | 34 (26, 42) | 140 (97, 182) | 0.12 (0.09, 0.15) | 0.18 (0.13, 0.24) | 1.49 (1.31, 1.66) | 7242 (4417, 10066) | 29590 (15513, 43666) | 19.05 (11.18, 26.91) | 32.66 (17.44, 47.88) | 1.72 (1.52, 1.92) |
| Poland | 91 (74, 108) | 255 (205, 305) | 0.22 (0.18, 0.25) | 0.35 (0.29, 0.41) | 1.48 (1.40, 1.56) | 14702 (9093, 20310) | 49848 (28550, 71146) | 34.75 (21.72, 47.79) | 75.85 (45.00, 106.70) | 2.53 (2.40, 2.66) |
| Portugal | 41 (31, 51) | 106 (82, 130) | 0.31 (0.24, 0.39) | 0.37 (0.30, 0.45) | 0.28 (0.00, 0.56) | 6836 (3916, 9755) | 23861 (14164, 33557) | 53.84 (31.26, 76.43) | 104.23 (64.31, 144.15) | 1.91 (1.52, 2.31) |
| Puerto Rico | 12 (8, 17) | 37 (25, 50) | 0.36 (0.25, 0.48) | 0.50 (0.34, 0.66) | 0.91 (0.67, 1.15) | 2424 (1297, 3551) | 9302 (4841, 13762) | 68.96 (37.20, 100.72) | 157.34 (83.62, 231.05) | 2.59 (2.33, 2.85) |
| Qatar | 0 (0, 1) | 6 (4, 8) | 0.54 (0.29, 0.78) | 0.96 (0.58, 1.34) | 1.91 (1.17, 2.67) | 98 (52, 144) | 2724 (1143, 4305) | 70.45 (32.96, 107.95) | 208.11 (96.89, 319.32) | 3.83 (3.23, 4.43) |
| Republic of Korea | 61 (47, 75) | 352 (279, 426) | 0.23 (0.18, 0.29) | 0.38 (0.30, 0.46) | 1.45 (0.74, 2.15) | 13749 (8171, 19327) | 112820 (66932, 158709) | 41.61 (24.60, 58.63) | 130.19 (78.49, 181.88) | 3.83 (3.01, 4.64) |
| Republic of Moldova | 6 (5, 7) | 12 (10, 15) | 0.15 (0.12, 0.17) | 0.21 (0.17, 0.25) | 0.62 (0.14, 1.11) | 1134 (685, 1582) | 2543 (1461, 3625) | 25.77 (15.78, 35.75) | 46.20 (27.30, 65.11) | 1.29 (0.73, 1.86) |
| Romania | 43 (35, 50) | 109 (89, 129) | 0.17 (0.14, 0.19) | 0.30 (0.24, 0.35) | 1.71 (1.58, 1.85) | 7031 (4250, 9813) | 21432 (12476, 30388) | 26.95 (16.53, 37.38) | 65.72 (39.06, 92.37) | 2.81 (2.67, 2.96) |
| Russian Federation | 344 (289, 399) | 988 (800, 1176) | 0.20 (0.17, 0.24) | 0.42 (0.35, 0.50) | 1.80 (1.35, 2.26) | 61620 (38421, 84819) | 206807 (124491, 289123) | 36.78 (23.00, 50.56) | 94.56 (58.93, 130.18) | 2.57 (2.07, 3.07) |
| Rwanda | 4 (3, 6) | 10 (6, 13) | 0.14 (0.09, 0.18) | 0.18 (0.11, 0.25) | -0.46 (-1.81, 0.90) | 798 (429, 1167) | 1655 (810, 2500) | 19.04 (10.53, 27.55) | 22.80 (11.01, 34.59) | -0.99 (-2.82, 0.88) |
| Saint Kitts and Nevis | 0 (0, 0) | 0 (0, 0) | 0.28 (0.20, 0.36) | 0.44 (0.31, 0.57) | 1.71 (1.42, 2.01) | 16 (9, 23) | 52 (28, 77) | 44.05 (25.12, 62.97) | 77.79 (43.37, 112.21) | 1.99 (1.73, 2.24) |
| Saint Lucia | 0 (0, 0) | 1 (1, 1) | 0.37 (0.25, 0.48) | 0.40 (0.26, 0.53) | -0.65 (-0.92, -0.37) | 48 (26, 70) | 172 (90, 253) | 55.72 (29.82, 81.61) | 74.97 (40.02, 109.93) | 0.28 (0.07, 0.50) |
| Saint Vincent and the Grenadines | 0 (0, 0) | 1 (0, 1) | 0.34 (0.22, 0.46) | 0.41 (0.28, 0.54) | 0.29 (0.09, 0.50) | 39 (22, 56) | 102 (58, 146) | 53.40 (29.49, 77.31) | 76.03 (43.78, 108.27) | 0.77 (0.61, 0.94) |
| Samoa | 0 (0, 0) | 0 (0, 1) | 0.23 (0.12, 0.34) | 0.36 (0.20, 0.52) | 1.33 (1.20, 1.45) | 32 (13, 50) | 93 (40, 145) | 34.58 (13.97, 55.20) | 61.64 (27.38, 95.90) | 1.69 (1.56, 1.82) |
| San Marino | 0 (0, 0) | 0 (0, 0) | 0.38 (0.28, 0.48) | 0.23 (0.13, 0.33) | -0.82 (-1.18, -0.45) | 30 (16, 44) | 50 (22, 78) | 91.84 (49.84, 133.84) | 71.77 (31.53, 112.02) | -0.14 (-0.52, 0.24) |
| Sao Tome and Principe | 0 (0, 0) | 0 (0, 0) | 0.13 (0.09, 0.16) | 0.24 (0.18, 0.30) | 2.15 (2.07, 2.24) | 14 (8, 21) | 49 (27, 71) | 17.88 (9.98, 25.78) | 37.59 (21.78, 53.40) | 2.44 (2.33, 2.54) |
| Saudi Arabia | 9 (7, 12) | 90 (63, 117) | 0.15 (0.10, 0.20) | 0.47 (0.34, 0.59) | 3.90 (3.79, 4.01) | 1953 (1023, 2884) | 34906 (15333, 54478) | 22.56 (11.60, 33.52) | 109.29 (57.21, 161.37) | 5.64 (5.48, 5.80) |
| Senegal | 4 (3, 5) | 13 (8, 19) | 0.11 (0.07, 0.15) | 0.19 (0.11, 0.27) | 1.58 (1.47, 1.68) | 621 (356, 886) | 2310 (1154, 3466) | 14.65 (7.78, 21.52) | 26.62 (12.62, 40.62) | 1.72 (1.61, 1.83) |
| Serbia | 24 (18, 30) | 70 (51, 88) | 0.26 (0.20, 0.32) | 0.41 (0.30, 0.52) | 1.53 (1.34, 1.72) | 3821 (2208, 5434) | 13316 (7118, 19514) | 36.71 (21.78, 51.65) | 85.71 (47.28, 124.14) | 2.74 (2.45, 3.04) |
| Seychelles | 0 (0, 0) | 0 (0, 0) | 0.13 (0.10, 0.16) | 0.23 (0.16, 0.29) | 2.01 (1.81, 2.21) | 15 (9, 22) | 60 (35, 85) | 26.23 (15.21, 37.26) | 49.71 (29.18, 70.24) | 2.40 (2.22, 2.57) |
| Sierra Leone | 3 (1, 4) | 7 (4, 10) | 0.10 (0.07, 0.14) | 0.17 (0.11, 0.23) | 1.51 (1.36, 1.66) | 367 (177, 557) | 1149 (541, 1758) | 13.01 (6.82, 19.20) | 22.77 (10.69, 34.84) | 1.44 (1.07, 1.81) |
| Singapore | 5 (4, 6) | 14 (11, 16) | 0.23 (0.18, 0.28) | 0.16 (0.14, 0.19) | -1.09 (-1.51, -0.67) | 1210 (720, 1699) | 5027 (3022, 7033) | 50.01 (29.28, 70.74) | 60.85 (37.06, 84.64) | 0.65 (0.18, 1.12) |
| Slovakia | 15 (12, 18) | 29 (22, 35) | 0.26 (0.21, 0.30) | 0.30 (0.24, 0.37) | 0.67 (0.61, 0.73) | 2582 (1530, 3635) | 6135 (3573, 8697) | 43.94 (26.26, 61.61) | 69.89 (41.41, 98.38) | 1.71 (1.62, 1.81) |
| Slovenia | 8 (6, 9) | 21 (17, 25) | 0.32 (0.27, 0.38) | 0.43 (0.35, 0.52) | 0.53 (0.36, 0.70) | 1477 (906, 2047) | 4676 (2790, 6562) | 61.81 (38.09, 85.53) | 113.00 (68.79, 157.21) | 1.81 (1.61, 2.02) |
| Solomon Islands | 0 (0, 0) | 1 (0, 1) | 0.15 (0.07, 0.23) | 0.24 (0.13, 0.36) | 1.54 (1.44, 1.64) | 35 (11, 60) | 161 (66, 255) | 21.77 (6.92, 36.61) | 38.75 (16.47, 61.04) | 1.80 (1.68, 1.93) |
| Somalia | 4 (2, 5) | 8 (5, 11) | 0.12 (0.07, 0.16) | 0.12 (0.08, 0.17) | 1.08 (-0.32, 2.49) | 674 (294, 1053) | 1647 (895, 2399) | 15.51 (7.59, 23.44) | 16.46 (8.76, 24.15) | 1.10 (-0.40, 2.63) |
| South Africa | 41 (29, 53) | 172 (116, 228) | 0.19 (0.13, 0.25) | 0.40 (0.27, 0.54) | 2.83 (2.65, 3.00) | 8317 (4606, 12029) | 33411 (18515, 48308) | 32.39 (17.50, 47.28) | 68.21 (37.57, 98.85) | 2.80 (2.64, 2.96) |
| South Sudan | 4 (2, 6) | 8 (5, 11) | 0.14 (0.08, 0.19) | 0.20 (0.12, 0.27) | 0.98 (0.75, 1.21) | 578 (277, 879) | 1374 (737, 2010) | 16.81 (8.64, 24.98) | 26.11 (13.62, 38.60) | 1.17 (0.83, 1.52) |
| Spain | 245 (183, 307) | 393 (307, 478) | 0.46 (0.35, 0.57) | 0.34 (0.28, 0.40) | -0.99 (-1.16, -0.82) | 43530 (26624, 60436) | 94765 (55194, 134337) | 85.29 (52.50, 118.08) | 101.71 (62.17, 141.24) | 0.35 (0.15, 0.55) |
| Sri Lanka | 20 (14, 27) | 108 (55, 161) | 0.21 (0.14, 0.28) | 0.42 (0.22, 0.62) | 3.16 (2.79, 3.53) | 4344 (2327, 6361) | 23514 (8940, 38088) | 33.84 (18.33, 49.35) | 88.64 (35.52, 141.77) | 3.94 (3.56, 4.33) |
| Sudan | 10 (6, 14) | 27 (17, 37) | 0.08 (0.06, 0.10) | 0.14 (0.09, 0.19) | 1.95 (1.78, 2.12) | 1717 (937, 2496) | 5744 (2984, 8504) | 11.78 (6.66, 16.91) | 22.74 (11.98, 33.51) | 2.25 (2.02, 2.48) |
| Suriname | 0 (0, 1) | 1 (1, 2) | 0.16 (0.11, 0.21) | 0.22 (0.13, 0.31) | 1.30 (1.10, 1.50) | 78 (45, 112) | 269 (142, 396) | 27.81 (15.43, 40.19) | 43.14 (23.01, 63.28) | 1.62 (1.41, 1.84) |
| Sweden | 58 (46, 69) | 90 (70, 109) | 0.36 (0.30, 0.43) | 0.36 (0.29, 0.43) | 0.20 (-0.02, 0.41) | 12186 (7413, 16958) | 21584 (12654, 30514) | 91.24 (56.00, 126.48) | 109.15 (65.27, 153.03) | 0.82 (0.54, 1.09) |
| Switzerland | 50 (39, 61) | 73 (57, 89) | 0.45 (0.36, 0.55) | 0.35 (0.28, 0.41) | -0.83 (-1.02, -0.65) | 10336 (6254, 14419) | 17674 (10762, 24587) | 104.69 (63.73, 145.65) | 100.52 (63.01, 138.03) | -0.10 (-0.38, 0.19) |
| Syrian Arab Republic | 6 (5, 8) | 30 (19, 41) | 0.12 (0.08, 0.15) | 0.27 (0.17, 0.36) | 3.76 (3.11, 4.42) | 1209 (703, 1715) | 6583 (3288, 9879) | 17.72 (9.95, 25.48) | 49.16 (25.14, 73.18) | 5.82 (4.61, 7.04) |
| Taiwan (Province of China) | 49 (35, 63) | 188 (140, 235) | 0.33 (0.24, 0.43) | 0.44 (0.33, 0.55) | 0.36 (-0.07, 0.80) | 11972 (6785, 17159) | 54740 (32501, 76980) | 69.51 (39.82, 99.21) | 137.56 (82.89, 192.24) | 1.74 (1.26, 2.23) |
| Tajikistan | 2 (2, 3) | 5 (3, 7) | 0.08 (0.06, 0.10) | 0.09 (0.06, 0.12) | -0.46 (-0.99, 0.07) | 423 (260, 587) | 983 (482, 1483) | 12.62 (7.51, 17.72) | 13.90 (6.90, 20.91) | -0.96 (-1.79, -0.11) |
| Thailand | 45 (34, 57) | 305 (196, 415) | 0.13 (0.09, 0.17) | 0.29 (0.19, 0.39) | 1.95 (1.67, 2.24) | 12205 (6971, 17440) | 86838 (43105, 130572) | 27.56 (15.80, 39.33) | 89.98 (44.34, 135.62) | 3.22 (2.85, 3.59) |
| Timor-Leste | 0 (0, 0) | 1 (1, 1) | 0.07 (0.04, 0.09) | 0.11 (0.07, 0.15) | 1.72 (1.34, 2.10) | 52 (24, 81) | 170 (82, 259) | 10.24 (5.52, 14.96) | 17.92 (8.59, 27.24) | 1.72 (1.03, 2.40) |
| Togo | 2 (1, 2) | 6 (3, 8) | 0.11 (0.07, 0.14) | 0.18 (0.11, 0.24) | 1.47 (1.41, 1.54) | 275 (159, 392) | 1199 (575, 1823) | 14.81 (7.88, 21.73) | 25.95 (12.57, 39.33) | 1.72 (1.63, 1.82) |
| Tokelau | 0 (0, 0) | 0 (0, 0) | 0.20 (0.10, 0.30) | 0.39 (0.23, 0.56) | 2.09 (1.99, 2.19) | 0 (0, 1) | 1 (0, 2) | 29.40 (11.03, 47.77) | 70.40 (34.15, 106.65) | 2.63 (2.52, 2.74) |
| Tonga | 0 (0, 0) | 0 (0, 1) | 0.28 (0.15, 0.41) | 0.45 (0.25, 0.66) | 1.48 (1.34, 1.61) | 26 (11, 40) | 63 (27, 99) | 44.31 (18.68, 69.93) | 76.86 (32.97, 120.75) | 1.63 (1.51, 1.76) |
| Trinidad and Tobago | 3 (2, 5) | 10 (5, 15) | 0.44 (0.27, 0.60) | 0.55 (0.30, 0.80) | 0.74 (0.60, 0.88) | 621 (293, 949) | 2139 (947, 3330) | 73.23 (34.59, 111.87) | 114.96 (52.72, 177.21) | 1.48 (1.33, 1.62) |
| Tunisia | 6 (5, 7) | 36 (21, 51) | 0.12 (0.09, 0.16) | 0.29 (0.17, 0.41) | 2.86 (2.76, 2.96) | 1080 (646, 1515) | 8273 (4209, 12337) | 19.18 (11.20, 27.17) | 62.59 (32.40, 92.78) | 3.94 (3.84, 4.04) |
| Türkiye | 53 (39, 67) | 314 (217, 410) | 0.17 (0.11, 0.22) | 0.36 (0.25, 0.47) | 3.17 (2.82, 3.52) | 8795 (5456, 12133) | 67017 (36523, 97511) | 22.90 (13.60, 32.19) | 72.53 (39.98, 105.08) | 4.43 (4.04, 4.83) |
| Turkmenistan | 2 (2, 2) | 7 (5, 10) | 0.09 (0.07, 0.11) | 0.19 (0.12, 0.25) | 2.04 (1.76, 2.33) | 360 (222, 498) | 1631 (792, 2471) | 15.09 (9.09, 21.10) | 35.44 (17.54, 53.35) | 2.51 (2.20, 2.81) |
| Tuvalu | 0 (0, 0) | 0 (0, 0) | 0.18 (0.09, 0.27) | 0.34 (0.20, 0.49) | 1.94 (1.74, 2.15) | 2 (1, 3) | 6 (2, 10) | 27.25 (11.43, 43.08) | 56.96 (24.56, 89.36) | 2.26 (2.05, 2.46) |
| Uganda | 8 (4, 11) | 25 (17, 33) | 0.11 (0.06, 0.15) | 0.18 (0.11, 0.24) | 1.34 (1.22, 1.46) | 1184 (596, 1771) | 4588 (2373, 6803) | 13.25 (6.31, 20.20) | 23.87 (11.72, 36.02) | 1.56 (1.42, 1.70) |
| Ukraine | 130 (109, 152) | 154 (101, 207) | 0.19 (0.16, 0.23) | 0.21 (0.14, 0.27) | -0.23 (-0.49, 0.02) | 23945 (14898, 32992) | 29371 (15138, 43604) | 37.40 (23.31, 51.48) | 43.83 (23.27, 64.39) | 0.08 (-0.26, 0.41) |
| United Arab Emirates | 1 (1, 1) | 9 (6, 12) | 0.24 (0.14, 0.34) | 0.41 (0.25, 0.58) | 3.56 (2.94, 4.18) | 253 (123, 382) | 2925 (1370, 4481) | 35.64 (15.98, 55.30) | 62.73 (32.88, 92.58) | 3.10 (2.62, 3.58) |
| United Kingdom | 289 (235, 342) | 461 (374, 548) | 0.31 (0.26, 0.37) | 0.32 (0.27, 0.38) | 0.06 (-0.15, 0.26) | 58940 (35710, 82169) | 111202 (68270, 154134) | 70.85 (43.26, 98.44) | 94.10 (59.33, 128.87) | 1.01 (0.81, 1.22) |
| United Republic of Tanzania | 15 (9, 21) | 40 (28, 53) | 0.13 (0.09, 0.17) | 0.17 (0.11, 0.22) | 0.60 (0.50, 0.70) | 2385 (1392, 3378) | 7213 (3889, 10537) | 16.45 (9.80, 23.10) | 23.13 (12.15, 34.10) | 0.78 (0.67, 0.88) |
| United States of America | 1169 (938, 1400) | 2488 (2051, 2924) | 0.36 (0.29, 0.43) | 0.42 (0.35, 0.49) | 0.12 (-0.19, 0.43) | 303306 (183431, 423181) | 783356 (489187, 1077525) | 100.26 (60.97, 139.56) | 154.77 (96.97, 212.56) | 1.01 (0.67, 1.35) |
| United States Virgin Islands | 0 (0, 0) | 0 (0, 1) | 0.29 (0.19, 0.39) | 0.28 (0.18, 0.37) | -0.29 (-0.54, -0.03) | 45 (24, 67) | 93 (50, 136) | 52.41 (26.77, 78.04) | 68.22 (36.99, 99.45) | 0.84 (0.61, 1.07) |
| Uruguay | 7 (6, 9) | 16 (13, 19) | 0.19 (0.15, 0.23) | 0.27 (0.22, 0.33) | 1.05 (0.93, 1.16) | 1374 (816, 1931) | 3629 (2139, 5119) | 37.73 (22.62, 52.83) | 72.54 (43.29, 101.78) | 2.00 (1.87, 2.13) |
| Uzbekistan | 11 (9, 14) | 48 (32, 64) | 0.09 (0.07, 0.11) | 0.19 (0.13, 0.25) | 2.80 (2.49, 3.11) | 2097 (1273, 2922) | 9779 (4689, 14869) | 15.19 (8.99, 21.39) | 33.04 (16.61, 49.46) | 2.75 (2.44, 3.06) |
| Vanuatu | 0 (0, 0) | 0 (0, 0) | 0.16 (0.08, 0.25) | 0.22 (0.11, 0.32) | 0.88 (0.84, 0.93) | 17 (7, 27) | 65 (25, 104) | 23.34 (8.50, 38.18) | 33.64 (13.03, 54.25) | 1.05 (0.98, 1.11) |
| Venezuela (Bolivarian Republic of) | 17 (13, 22) | 94 (56, 132) | 0.18 (0.12, 0.23) | 0.33 (0.19, 0.46) | 1.79 (1.56, 2.01) | 3927 (2253, 5602) | 23301 (10887, 35714) | 34.16 (18.66, 49.67) | 78.42 (37.13, 119.72) | 2.53 (2.28, 2.77) |
| Viet Nam | 55 (34, 76) | 322 (219, 425) | 0.14 (0.09, 0.20) | 0.36 (0.24, 0.48) | 3.21 (3.15, 3.28) | 9489 (4730, 14247) | 71368 (34470, 108267) | 21.43 (10.37, 32.49) | 69.62 (34.88, 104.35) | 4.10 (4.02, 4.18) |
| Yemen | 5 (3, 7) | 21 (13, 29) | 0.07 (0.05, 0.10) | 0.13 (0.08, 0.18) | 2.13 (1.97, 2.28) | 836 (406, 1267) | 5018 (2202, 7834) | 10.39 (4.99, 15.79) | 22.24 (11.42, 33.06) | 2.87 (2.58, 3.17) |
| Zambia | 4 (2, 6) | 13 (8, 18) | 0.13 (0.09, 0.18) | 0.20 (0.12, 0.27) | 1.09 (0.94, 1.25) | 690 (389, 991) | 2611 (1300, 3922) | 17.00 (9.34, 24.66) | 28.22 (14.64, 41.79) | 1.40 (1.21, 1.58) |
| Zimbabwe | 6 (4, 8) | 14 (9, 19) | 0.15 (0.10, 0.21) | 0.21 (0.14, 0.29) | 0.99 (0.49, 1.49) | 1029 (531, 1527) | 2767 (1425, 4108) | 20.94 (10.58, 31.30) | 31.85 (16.60, 47.10) | 1.17 (0.57, 1.76) |
