# Supplementary material for: Global, regional, and national burden of bone and joint infections, 1990–2021: a comprehensive analysis of trends, pathogens, and antimicrobial resistance
Source: Front Cell Infect Microbiol. 2026 Jun 2;16:1858745. doi: 10.3389/fcimb.2026.1858745 (PMC13269380; doi:10.3389/fcimb.2026.1858745)
Supplement: Supplementary file 7 [file Table2.docx]

Table S2 The distribution of global mortality and disability-adjusted life years by age for osteoarticular infections in 2021

|  | Mortality | | DALYs | |
| --- | --- | --- | --- | --- |
| Age(years) | Number | Rate | Number | Rate |
| Under 5 | 156 (56, 256) | 0.02 (0.01, 0.04) | 13908 (5023, 22793) | 2.11 (0.76, 3.46) |
| 5 to 9 | 38 (9, 66) | 0.01 (0.00, 0.01) | 10139 (2068, 18211) | 1.48 (0.30, 2.65) |
| 10 to 14 | 54 (25, 83) | 0.01 (0.00, 0.01) | 21250 (7486, 35014) | 3.19 (1.12, 5.25) |
| 15 to 19 | 86 (46, 127) | 0.01 (0.01, 0.02) | 46334 (19531, 73138) | 7.43 (3.13, 11.72) |
| 20 to 24 | 100 (55, 145) | 0.02 (0.01, 0.02) | 73627 (29648, 117605) | 12.33 (4.96, 19.69) |
| 25 to 29 | 124 (68, 180) | 0.02 (0.01, 0.03) | 111191 (37401, 184982) | 18.90 (6.36, 31.44) |
| 30 to 34 | 190 (113, 268) | 0.03 (0.02, 0.04) | 172450 (68130, 276769) | 28.53 (11.27, 45.79) |
| 35 to 39 | 264 (147, 381) | 0.05 (0.03, 0.07) | 215927 (78129, 353726) | 38.50 (13.93, 63.07) |
| 40 to 44 | 379 (222, 536) | 0.08 (0.04, 0.11) | 303019 (110017, 496022) | 60.57 (21.99, 99.15) |
| 45 to 49 | 654 (359, 948) | 0.14 (0.08, 0.20) | 545829 (203770, 887889) | 115.27 (43.03, 187.51) |
| 50 to 54 | 1095 (480, 1709) | 0.25 (0.11, 0.38) | 343944 (88415, 599472) | 77.30 (19.87, 134.74) |
| 55 to 59 | 1589 (618, 2560) | 0.40 (0.16, 0.65) | 511210 (175796, 846623) | 129.18 (44.42, 213.94) |
| 60 to 64 | 2183 (774, 3593) | 0.68 (0.24, 1.12) | 698971 (115895, 1282047) | 218.40 (36.21, 400.58) |
| 65 to 69 | 2882 (1378, 4386) | 1.04 (0.50, 1.59) | 937941 (285964, 1589918) | 340.03 (103.67, 576.39) |
| 70 to 74 | 3373 (1629, 5117) | 1.64 (0.79, 2.49) | 477312 (201448, 753177) | 231.89 (97.87, 365.91) |
| 75 to 79 | 3220 (1621, 4819) | 2.44 (1.23, 3.65) | 444968 (197945, 691992) | 337.39 (150.09, 524.70) |
| 80 to 84 | 3197 (1416, 4979) | 3.65 (1.62, 5.68) | 431455 (132978, 729933) | 492.62 (151.83, 833.42) |
| 85 to 89 | 2745 (1190, 4300) | 6.00 (2.60, 9.40) | 378513 (132261, 624765) | 827.86 (289.27, 1366.45) |
| 90 to 94 | 1573 (629, 2518) | 8.80 (3.51, 14.08) | 227011 (37978, 416044) | 1268.97 (212.30, 2325.65) |
| 95 plus | 603 (229, 977) | 11.07 (4.20, 17.93) | 89610 (15633, 163587) | 1644.13 (286.82, 3001.44) |
