# Supplementary material for: Global, regional, and national burden of bone and joint infections, 1990–2021: a comprehensive analysis of trends, pathogens, and antimicrobial resistance
Source: Front Cell Infect Microbiol. 2026 Jun 2;16:1858745. doi: 10.3389/fcimb.2026.1858745 (PMC13269380; doi:10.3389/fcimb.2026.1858745)
Supplement: Supplementary file 8 [file Table3.docx]

Table S3 Osteoarticular infections mortality and DALYs attributable to pathogens in 1990 and 2021 for both sexes and estimated annual percentage change in age-standardized rates by pathogens

| Pathogens | Deaths | | | | | DALYs | | | | |
| --- | --- | --- | --- | --- | --- | --- | --- | --- | --- | --- |
|  | All ages number in 1990 (95% UI) | All ages number in 2021 (95% UI) | Age-standardized rate in 1990 (95% UI) | Age-standardized rate in 2021 (95% UI) | Estimated annual percentage change (95% CI),1990-2021 | All ages number in 1990 (95% UI) | All ages number in 2021 (95% UI) | Age-standardized rate in 1990 (95% UI) | Age-standardized rate in 2021 (95% UI) | Estimated annual percentage change (95% CI),1990-2021 |
| **Gram-positive bacteria** |  |  |  |  |  |  |  |  |  |  |
| *Enterococcus faecalis* | 210 (167, 253) | 848 (677, 1018) | 0.01 (0.00, 0.01) | 0.01 (0.01, 0.01) | 1.70 (1.61, 1.79) | 51111 (30649, 71572) | 234511 (140535, 328488) | 1.30 (0.77, 1.82) | 2.77 (1.67, 3.88) | 2.42 (2.29, 2.56) |
| *Enterococcus faecium* | 99 (77, 122) | 480 (379, 581) | 0.00 (0.00, 0.00) | 0.01 (0.00, 0.01) | 2.18 (2.01, 2.35) | 22087 (13313, 30860) | 122075 (73133, 171017) | 0.59 (0.35, 0.83) | 1.45 (0.87, 2.04) | 2.87 (2.64, 3.10) |
| *Group A Streptococcus* | 155 (120, 190) | 396 (314, 477) | 0.00 (0.00, 0.00) | 0.00 (0.00, 0.01) | 0.50 (0.45, 0.54) | 37088 (22858, 51319) | 111907 (68028, 155787) | 0.88 (0.54, 1.22) | 1.32 (0.81, 1.84) | 1.29 (1.23, 1.35) |
| *Group B Streptococcus* | 271 (215, 327) | 980 (782, 1178) | 0.01 (0.01, 0.01) | 0.01 (0.01, 0.01) | 1.33 (1.27, 1.39) | 62422 (38015, 86830) | 256845 (154812, 358879) | 1.60 (0.96, 2.23) | 3.03 (1.83, 4.24) | 2.04 (1.94, 2.15) |
| *Staphylococcus aureus* | 1383 (1131, 1635) | 4993 (4034, 5952) | 0.04 (0.03, 0.05) | 0.06 (0.05, 0.07) | 1.41 (1.35, 1.47) | 407379 (247198, 567560) | 1712839 (1022371, 2403308) | 10.10 (6.07, 14.13) | 20.18 (12.09, 28.26) | 2.22 (2.10, 2.33) |
| *Streptococcus pneumoniae* | 446 (349, 542) | 1012 (790, 1234) | 0.01 (0.01, 0.02) | 0.01 (0.01, 0.01) | -0.36 (-0.85, 0.14) | 74528 (46377, 102679) | 246923 (149458, 344388) | 1.97 (1.20, 2.73) | 2.95 (1.79, 4.11) | 1.12 (0.65, 1.60) |
| *Acinetobacter baumannii* | 91 (71, 111) | 288 (227, 348) | 0.00 (0.00, 0.00) | 0.00 (0.00, 0.00) | 1.00 (0.96, 1.05) | 21908 (13394, 30422) | 79153 (47763, 110544) | 0.54 (0.33, 0.76) | 0.94 (0.57, 1.30) | 1.74 (1.66, 1.82) |
| *Aeromonas spp.* | 26 (21, 31) | 100 (78, 121) | 0.00 (0.00, 0.00) | 0.00 (0.00, 0.00) | 1.54 (1.46, 1.61) | 6717 (4127, 9307) | 28719 (17172, 40266) | 0.17 (0.10, 0.23) | 0.34 (0.20, 0.48) | 2.28 (2.16, 2.40) |
| **Gram-negative bacteria** |  |  |  |  |  |  |  |  |  |  |
| *Burkholderia spp.* | 6 (5, 7) | 23 (18, 28) | 0.00 (0.00, 0.00) | 0.00 (0.00, 0.00) | 1.54 (1.46, 1.61) | 1521 (934, 2107) | 6502 (3888, 9116) | 0.04 (0.02, 0.05) | 0.08 (0.05, 0.11) | 2.28 (2.16, 2.40) |
| *Chlamydia spp.* | 7 (6, 8) | 26 (20, 32) | 0.00 (0.00, 0.00) | 0.00 (0.00, 0.00) | 1.54 (1.46, 1.61) | 1755 (1078, 2431) | 7502 (4486, 10518) | 0.04 (0.03, 0.06) | 0.09 (0.05, 0.12) | 2.28 (2.16, 2.40) |
| *Citrobacter spp.* | 53 (43, 64) | 202 (158, 246) | 0.00 (0.00, 0.00) | 0.00 (0.00, 0.00) | 1.54 (1.46, 1.61) | 13589 (8348, 18830) | 58102 (34741, 81463) | 0.34 (0.21, 0.47) | 0.69 (0.41, 0.96) | 2.28 (2.16, 2.40) |
| *Enterobacter spp.* | 294 (238, 350) | 967 (782, 1152) | 0.01 (0.01, 0.01) | 0.01 (0.01, 0.01) | 1.17 (1.12, 1.23) | 61194 (37525, 84862) | 229193 (138795, 319591) | 1.48 (0.90, 2.06) | 2.70 (1.64, 3.76) | 1.93 (1.83, 2.02) |
| *Escherichia coli* | 871 (700, 1041) | 2696 (2137, 3255) | 0.02 (0.02, 0.03) | 0.03 (0.03, 0.04) | 0.85 (0.81, 0.89) | 158766 (98496, 219036) | 557041 (337315, 776767) | 4.03 (2.47, 5.58) | 6.58 (4.00, 9.16) | 1.55 (1.49, 1.60) |
| *Haemophilus influenzae* | 29 (23, 34) | 109 (85, 132) | 0.00 (0.00, 0.00) | 0.00 (0.00, 0.00) | 1.54 (1.46, 1.61) | 7322 (4498, 10145) | 31304 (18718, 43891) | 0.18 (0.11, 0.25) | 0.37 (0.22, 0.52) | 2.28 (2.16, 2.40) |
| *Klebsiella pneumoniae* | 856 (678, 1033) | 2333 (1852, 2814) | 0.02 (0.02, 0.03) | 0.03 (0.02, 0.03) | 0.54 (0.50, 0.59) | 118100 (75322, 160879) | 357384 (221892, 492876) | 2.89 (1.82, 3.96) | 4.22 (2.63, 5.80) | 1.18 (1.13, 1.23) |
| *Morganella spp.* | 188 (146, 230) | 604 (481, 728) | 0.01 (0.00, 0.01) | 0.01 (0.01, 0.01) | 0.94 (0.90, 0.99) | 38751 (23491, 54012) | 143698 (85820, 201577) | 1.01 (0.60, 1.41) | 1.70 (1.02, 2.38) | 1.64 (1.57, 1.71) |
| *Neisseria meningitidis* | 24 (19, 28) | 89 (70, 109) | 0.00 (0.00, 0.00) | 0.00 (0.00, 0.00) | 1.54 (1.46, 1.61) | 6016 (3696, 8336) | 25721 (15379, 36063) | 0.15 (0.09, 0.21) | 0.30 (0.18, 0.43) | 2.28 (2.16, 2.40) |
| *Proteus spp.* | 408 (324, 492) | 1398 (1104, 1692) | 0.01 (0.01, 0.01) | 0.02 (0.01, 0.02) | 1.10 (1.06, 1.14) | 91116 (54960, 127273) | 359937 (212458, 507416) | 2.39 (1.42, 3.36) | 4.27 (2.52, 6.01) | 1.83 (1.76, 1.91) |
| *Pseudomonas aeruginosa* | 1252 (1008, 1495) | 3682 (2925, 4440) | 0.03 (0.03, 0.04) | 0.04 (0.04, 0.05) | 0.74 (0.70, 0.78) | 164009 (104975, 223042) | 533735 (329898, 737572) | 4.08 (2.57, 5.58) | 6.32 (3.92, 8.72) | 1.37 (1.32, 1.42) |
| *Serratia spp.* | 165 (130, 199) | 495 (391, 599) | 0.00 (0.00, 0.01) | 0.01 (0.00, 0.01) | 0.90 (0.86, 0.95) | 42294 (25046, 59542) | 144787 (85358, 204215) | 1.02 (0.60, 1.43) | 1.71 (1.01, 2.40) | 1.64 (1.57, 1.71) |
| **Fungi** |  |  |  |  |  |  |  |  |  |  |
| *Candida spp.* | 9 (8, 11) | 36 (28, 43) | 0.00 (0.00, 0.00) | 0.00 (0.00, 0.00) | 1.54 (1.46, 1.61) | 2398 (1473, 3323) | 10253 (6130, 14375) | 0.06 (0.04, 0.08) | 0.12 (0.07, 0.17) | 2.28 (2.16, 2.40) |
